# Bundle analytics, a computational framework for investigating the shapes and profiles of brain pathways across populations

**DOI:** 10.1038/s41598-020-74054-4

**Published:** 2020-10-13

**Authors:** Bramsh Qamar Chandio, Shannon Leigh Risacher, Franco Pestilli, Daniel Bullock, Fang-Cheng Yeh, Serge Koudoro, Ariel Rokem, Jaroslaw Harezlak, Eleftherios Garyfallidis

**Affiliations:** 1 Department of Intelligent Systems Engineering, Luddy School of Informatics, Computing and Engineering, Indiana University Bloomington, Bloomington, IN USA; 2 School of Public Health, Indiana University Bloomington, Bloomington, IN USA; 3 Department of Psychology, The University of Texas, Austin, TX USA; 4 Department of Psychological and Brain Sciences, Indiana University Bloomington, Bloomington, IN USA; 5 Department of Neurological Surgery, University of Pittsburgh, Pittsburgh, PA USA; 6 Department of Psychology and eScience Institute, University of Washington, Washington, DC USA; 7 Indiana University School of Medicine, Indianapolis, IN USA

**Keywords:** Computational neuroscience, Computational neuroscience, Data processing, Image processing, Software, Statistical methods, Parkinson's disease, Biomarkers, Medical research, Computational science, Computer science, Software, Statistics

## Abstract

Tractography has created new horizons for researchers to study brain connectivity in vivo. However, tractography is an advanced and challenging method that has not been used so far for medical data analysis at a large scale in comparison to other traditional brain imaging methods. This work allows tractography to be used for large scale and high-quality medical analytics. BUndle ANalytics (BUAN) is a fast, robust, and flexible computational framework for real-world tractometric studies. BUAN combines tractography and anatomical information to analyze the challenging datasets and identifies significant group differences in specific locations of the white matter bundles. Additionally, BUAN takes the shape of the bundles into consideration for the analysis. BUAN compares the shapes of the bundles using a metric called bundle adjacency which calculates shape similarity between two given bundles. BUAN builds networks of bundle shape similarities that can be paramount for automating quality control. BUAN is freely available in DIPY. Results are presented using publicly available Parkinson’s Progression Markers Initiative data.

## Introduction

The human brain contains billions of axons that bundle together in tracts and fasciculi. These can be reconstructed in vivo by collecting diffusion MRI data^1–3^ and deploying tractography algorithms^4–7^. The outputs of tractography algorithms are called tractograms. These tractograms are represented digitally using streamlines, which are representations of 3D curves traversing the brain. Whole-brain tractograms are densely populated with millions of streamlines which makes it difficult to visually and computationally inspect and characterize brain pathways. When streamlines of similar shapes and characteristics travel together through the white matter they are called bundles. These bundles approximate the white matter fiber bundles that connect distant parts of the brain to each other and are also known as tracts or fasciculi. These bundles of axons carry crucial information between cortical and/or subcortical areas and potential damage to these bundles, for example, from surgery, trauma, or disease can have tremendous consequences for the patient’s cognitive function and quality of life. Every bundle has a different functional association or set of functional associations. For example, the arcuate fasciculus is involved in the understanding of language^8^ while the optic radiation is involved primarily with visual processing^9,10^. Therefore, a valuable capability for the analysis of digital models of white matter is the ability to automatically identify known bundles contained within the whole brain tractograms. This process is known as bundle recognition^11^, extraction, or segmentation.

Manual virtual dissection^12–14^ and automatic bundle extraction^11,15–20^ of white matter bundles has enabled the scientific community to gather thousands of extracted fiber tract exemplars from source tractograms and visualize them in vivo. With the rise of machine learning in the field of neuroimaging, we are able to automate complex and unwieldy tasks such as white matter fiber bundle segmentation from whole-brain tractograms. Segmenting bundles has become convenient, efficient and fast^11,21^ and this practice has resulted in the generation of exceedingly large data sets^22–26^. With a great amount of data available to them, the neuroscience community can now proceed to perform statistically sophisticated group comparisons on the acquired tracts. In the past decade, a plethora of methods for analyzing tractograms and combining them with anatomical information have been developed^20,27–34^. These analytical methods combining tractography and anatomical measures such as fractional anisotropy (FA), mean diffusivity (MD), radial diffusivity (RD), and axial diffusivity (AD)^35,36^ to study pathways are termed as tractometric analysis methods^31^. Furthermore, tractometric analysis methods look at how bundles differ between specified groups. Recently, many studies^27–34,37^ have applied statistical methods to the study of group differences along the length of tracts. These are often called bundle profiles^28^.

In the field of neuroimaging, many different methods exist separately^27–34^ for the different tasks required to perform tractometric studies. Building a complete pipeline requires careful planning and forethought, especially for clinical applications. However, no such end-to-end pipeline has achieved wide acceptance or prominence which offers one platform to extract and perform group comparisons of white matter bundles.

In our work, we present BUndle ANalytics (BUAN), an end-to-end computational framework that can precisely extract bundles, perform statistical analyses across groups, and compare the shapes of different bundles. Our framework starts by taking a whole-brain target tractogram as input and then performs streamline-based registration of the tractogram to MNI space using an atlas of exemplar bundles (template). It next proceeds to bundle extraction, wherein it applies the auto-calibrated version of RecoBundles^11^ (see "Auto-calibration" section) which is sufficiently robust to permit the extraction of particularly long or short tract bundles. For each of the tracts extracted, BUAN generates a tract profile for each subject. BUAN then performs statistical analysis on the bundle profiles to discover group differences along the length of tracts based on anatomical measures such as fractional anisotropy (FA), mean diffusivity (MD), radial diffusivity (RD), axial diffusivity (AD)^35,36^, Constant Solid Angle-generalized fractional anisotropy (CSA-GFA)^38,39^, and Constant Solid Angle-quantitative anisotropy (CSA-QA)^40^. Indeed, BUAN is sufficiently robust such that a wide array of anatomical information can be integrated into the analysis. Group comparisons on bundle profiles are performed using linear mixed models^41–43^. BUAN is capable of precisely localizing group differences to specific subsections of bundles. BUAN has the further advantage of permitting shape analysis of extracted tracts. In this paper, we present a novel graph-theoretic approach to compare the shapes of two bundles of the same type using bundle adjacency (BA) method^21^. Bundle adjacency is a bounded method for expressing how similar the shapes of two bundles are and bundle adjacency score ranges from 0 to 1. We construct a network of bundles across the subjects and calculate bundle adjacency (BA) among them. This results in a network graph/adjacency matrix that gives insights about inter-group and intra-group shape differences. This method can be used for quality assurance and is performed in common ’atlas’ space. Furthermore, BUAN operates without the need for nonrigid deformations, a process that could potentially make it harder to interpret group differences.

Our fully automated, streamline based approach enables us to create a robust, fast, and flexible computational framework. It is important to note that our framework is applicable to every kind of diffusion brain image data including hard-to-process clinical data. It does not require any sort of heavy training that requires large amounts of data and nor does it depend on any training data set. BUAN is publicly available in DIPY^44^ through python scripts and command-line interfaces.

## Results

### Overview

**Figure 1:**
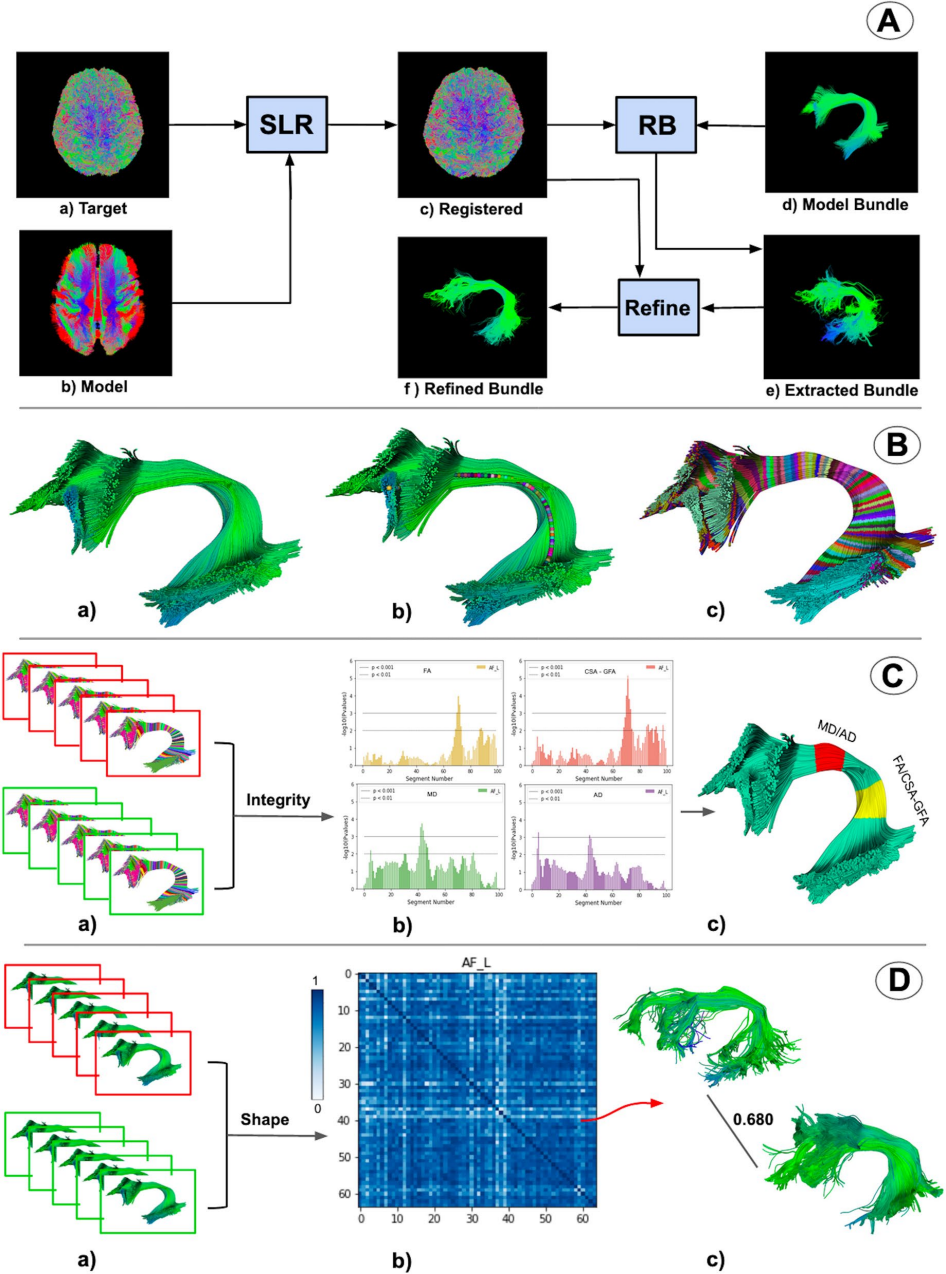
Overview of bundle analytics (BUAN) framework. (**A**) shows the process of bundle recognition. (**B**) shows the process of creating assignment maps (segments) on each extracted bundle. (**C**) shows the bundle profile analysis concept where groups (red and green) with assignment maps can be used to find statistically significant group differences at specific locations in the bundles. Notice in (**C**.c) how multiple metrics can be significant in one area (shown with red) and others can be significant in other areas (shown with yellow). Finally (**D**), shows an example of the network-based bundle shape similarity analysis method of BUAN. In (**D**.c), a single scalar value [0–1] summarizes the shape differences between the two bundles. The matrix (**D**.b) provides the complete information of all shape differences in the populations for the the left arcuate fasciculus.

Here, we provide an overview of BUAN. Fig. 1A, shows the process of bundle extraction. We registered our input tractogram [target tractogram (A.a)] to the model atlas (A.b) space using streamline-based linear registration (SLR)^45^. For extracting bundles from whole-brain tractograms, we have applied Recobundles (RB)^11^. RB takes the registered tractogram (A.c) and model bundle (A.d) as input and extracts bundle (A.e). A new auto-calibration step in RecoBundles (refer to "Auto-calibration" section for details) has been added for extracting small and difficult to find bundles. (A.f) shows the final extracted bundle after auto-calibration (refine). Fig. 1B, shows the next step in the process wherein BUAN segments the final extracted bundle: the left arcuate fasciculus (AF_L) in small segments. We call this step the assignment step because every point of the bundle is assigned to the closest point of the model centroid. In (B.a) we see a depiction of a given bundle, in (B.b) we see model bundle centroids projected on to the bundle (B.a). In (B.c), each point on streamline is assigned to the nearest centroid point (shown with random colors). In Fig. 1C, we see the tractometric part of our framework, where we provided extracted and segmented bundles from all subjects as input as shown in (C.a). Here, the red box indicates patient data while the green box indicates healthy control data. We found significant differences across populations using information from anatomical measures such as fractional anisotropy (FA), mean diffusivity (MD), radial diffusivity (RD), axial diffusivity (AD), generalized fractional anisotropy (GFA) and several other metrics. We applied linear mixed models (LMM)^41–43^ to find group differences across subjects for all bundles at specific locations. In (C.b) we see the exact segment where anatomical measures differed between groups (e.g., FA is different between patients and controls). Notice in (C.c) that FA and CSA-GFA differed at 65–75 segments while MD and AD differed at 45–55 segments as indicated by *p* value < 0.001. BUAN can locate and visualize exactly where a significant difference is found between the two populations. Now we can build a more complete picture of the data by looking into the shape differences. This is shown in Fig. 1D. In (D.a), we took extracted bundles from all subjects as input, once more, the red box indicates patient data while the green box indicates healthy control data (similar to Fig. 1C). However, this time the input was extracted bundles without any information associated with segments (assignment maps). Explicitly, we only provided the bundles themselves. The goal was thus to see the shape differences along the lengths of the bundles and study their sub-clusters. In (D.b), BUAN created a similarity matrix by calculating bundle adjacency (BA)^21^, our proposed shape similarity metric, between each subjects’ bundle and every other subjects’ bundle. Higher BA values (dark blue color) indicate higher shape similarity and lower BA values (light blue color) indicate lower shape similarity among bundles. Using shape similarity matrix information, we can go back and visually inspect the shape of the bundles and what BA score they were assigned. An example is shown in (D.c). Bundle adjacency is a bounded measure and takes values between 0 to 1, such that 0 means no shape similarity, i.e. no similar adjacent clusters of streamlines were found between the two bundles and 1 means maximum similarity, i.e. all clusters of both bundles had at least one neighbor.

In this work we used data from the publicly available Parkinson’s Progression Markers Initiative (PPMI) database^46^. In the following sections, we present results generated by BUAN on 30 extracted white matter fiber bundles for 64 subjects: 32 controls and 32 patients.

The complete description of the methods and data summarized here can be found in "Methods" section.

### Bundle profiles

**Figure 2:**
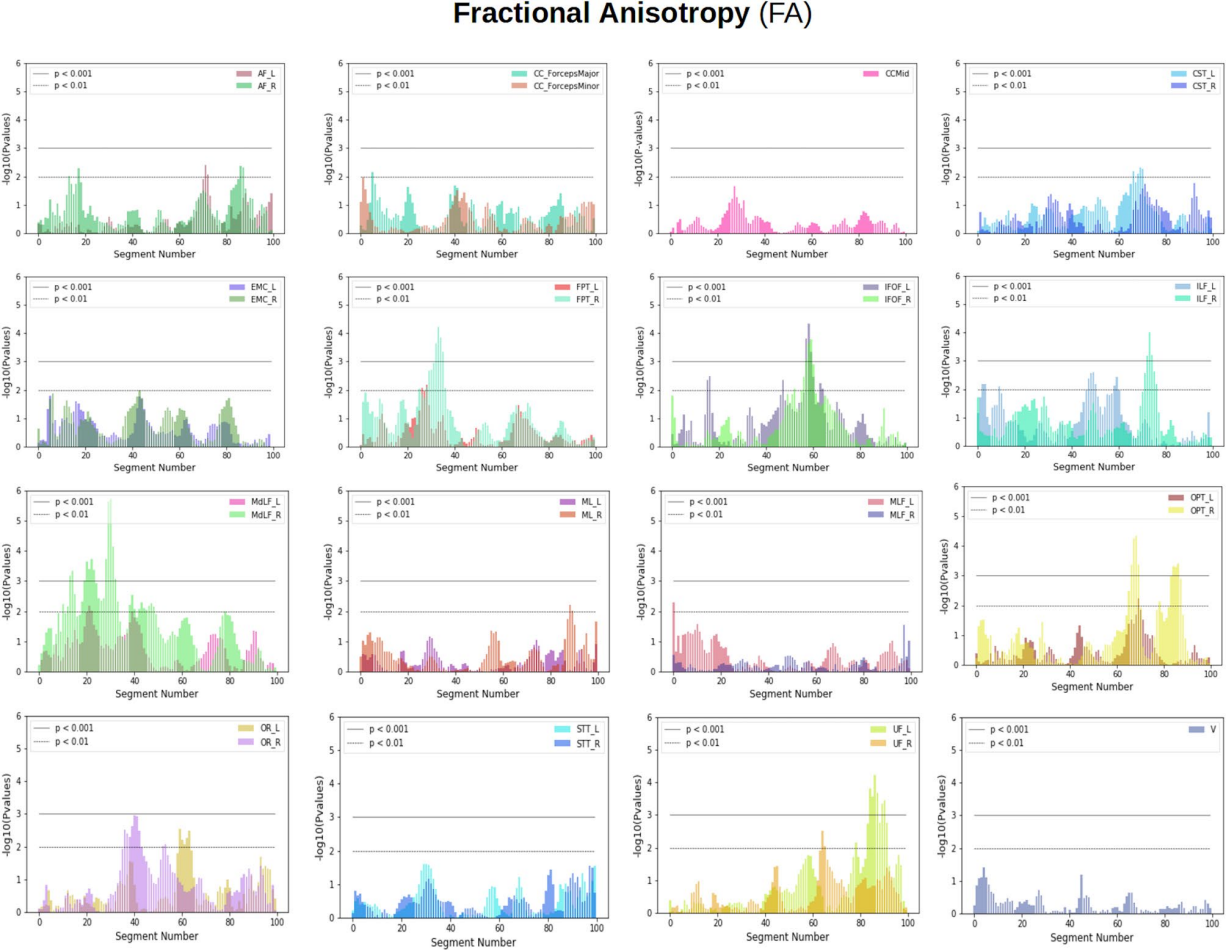
Showing plots summarizing population differences for fractional anisotropy (FA) using LMM for 30 bundles along their length. On the x-axis, we have segment numbers and on the y-axis, we have negative logarithms of *p* values. In the plots, the horizontal lower line indicates a *p* value < 0.01 and the horizontal upper line indicates a *p* value < 0.001. The *p* value at a specific segment implies how much significant FA group differences are there between patients and healthy controls for that particular bundle. Most plots show left and right bundles of the same type except V and CCMid. The plot at first row, second column shows simultaneously Minor and Major Forceps of CC. The rest of the plots show the left and right parts of the same type of bundle e.g. AF left (AF_L) vs AF right (AF_R). Notice for example that the IFOF bundles (left and right) are significantly different around segment 60.

Bundle profiles were generated for 30 extracted white matter bundles of all 64 subjects by creating assignment maps of extracted bundles. Each assignment map contained 100 segments per bundle. All were generated in common space. See "Assignment maps" section for details about the method. Data were then transformed back into native space to project anatomical measures, fractional anisotropy (FA), mean diffusivity (MD), radial diffusivity (RD), axial diffusivity (AD)^35,36^, Constant Solid Angle-generalized fractional anisotropy (CSA-GFA)^38,39^, and Constant Solid Angle-quantitative anisotropy (CSA-QA)^40^ on the segments of bundles. Throughout the process of bundle profile creation, no data was discarded. We did not smooth or deform our tracts or sub-sample them. We used all the points of each streamline and all the streamlines of the bundles. Comparisons between groups (patients vs controls) were done using linear mixed models (LMM) which adjusted for the correlations between the streamline data extracted from the same subject’s tract. Fig. 2 shows the analyses summary for the 100 segments for each tract. Information about the white matter tracts used in the study can be found in the supplementary materials, Appendix A.1 Fig. A1. Statistically significant differences (*p* value < 0.001) between the groups were found in FA along the IFOF_L and IFOF_R tracts between segments 55 and 65 as shown in Fig. 2. We also found group differences on FPT_R bundle at 30–37 segments, MdLF_R at 25–29 segments, and 30–35 segments, ILF_R at 72–78 segments, OPT_R 70–75, and 85–90, OR_L at 38–45 segments and finally for UF_L at 85–95 segments. The LMM result plots for other anatomical measures can be found in the supplementary materials Appendix A.2, for MD Fig. A4, RD Fig. A2, AD Fig. A3, CSA-GFA Fig. A5, and CSA-QA Fig. A6.

**Figure 3:**
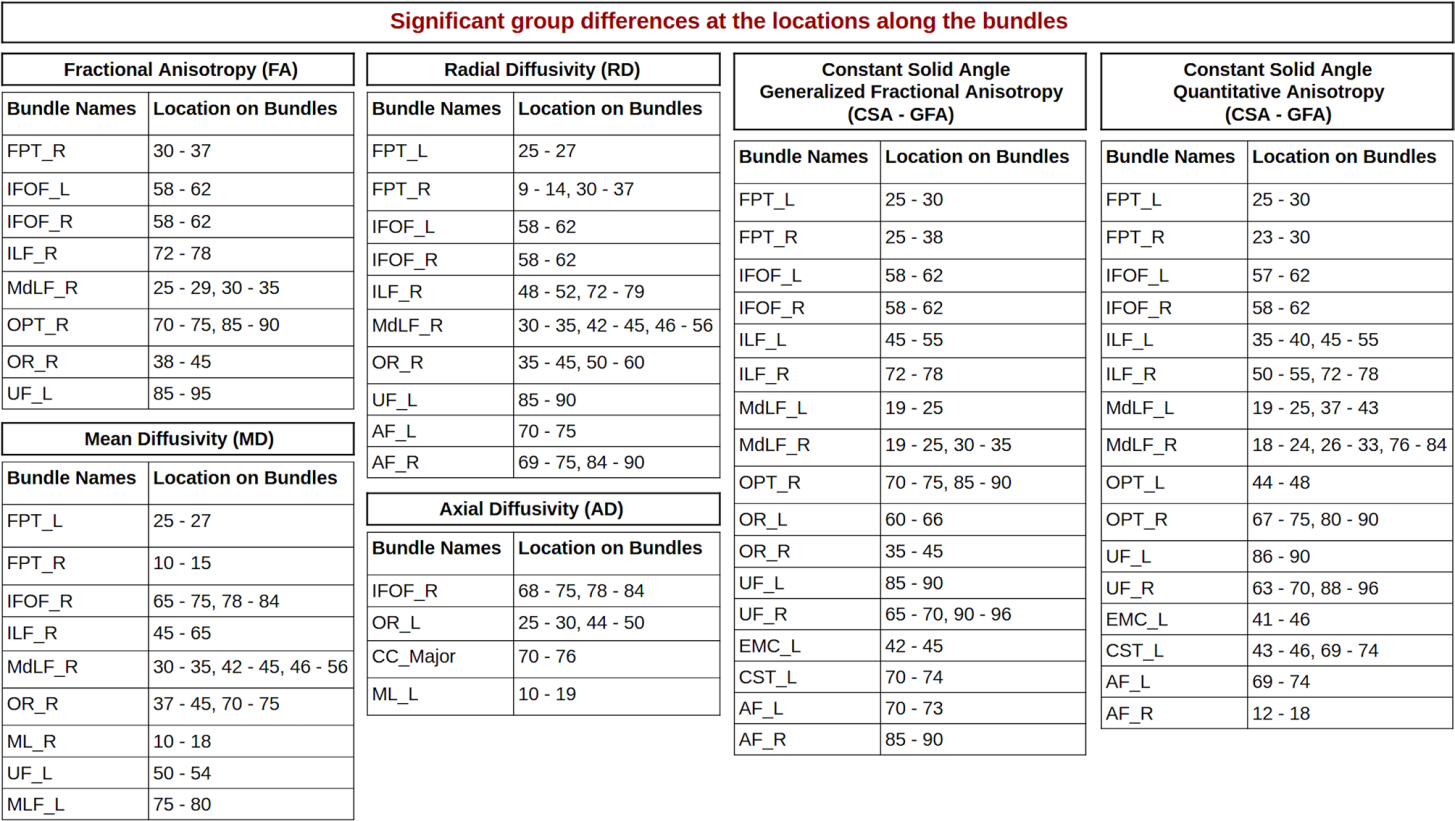
Summary tables showing locations with statistically significant differences in the bundles between groups for different metrics. Listed are tables for FA, MD, RD, AD, CSA-GFA, and CSA-QA. Each table has a bundle name on the left column and the right column has a location on a bundle where significant differences between patient and control data were identified by LMM. All locations have *p* values <0.001. Overall, we observed higher anisotropy and lower diffusivity for patients as compared to controls at these locations on the bundles.

We found group differences for FA, MD, RD, AD, CSA-GFA, and CSA-QA across different bundles. The LMM plots can be found in the supplementary materials in Appendix A.2. MD results are available in Sec. Appendix A.2 Fig.A4, RD in Sec. Appendix A.2 Fig. A2, AD in Sec. Appendix A.2 Fig.A3, CSA-GFA8 in Sec. Appendix A.2 Fig.A5, and CSA-QA in Sec. Appendix A.2 Fig.A6. The summarized version of results for all 6 anatomical measures is described in Fig. 3. The locations on the bundles are selected based on the criteria of *p* values < 0.001 for group differences at that location. We found consistency in the results for the same bundles with differences at similar locations for FA, RD, GFA, and QA metrics/measures. We also found results for MD, RD, and AD to have the same locations with significant differences. We examined the locations mentioned in Fig. 3 to find actual differences between patient data and control data at those locations. FA, CSA-GFA, and CSA-QA values increase in patients, the average anisotropy values of all these 3 metrics used here are higher in patient data than in healthy control data. AD, RD, and MD values decrease in patients, and the average diffusivity value in patients is lower than in healthy controls.

**Figure 4:**
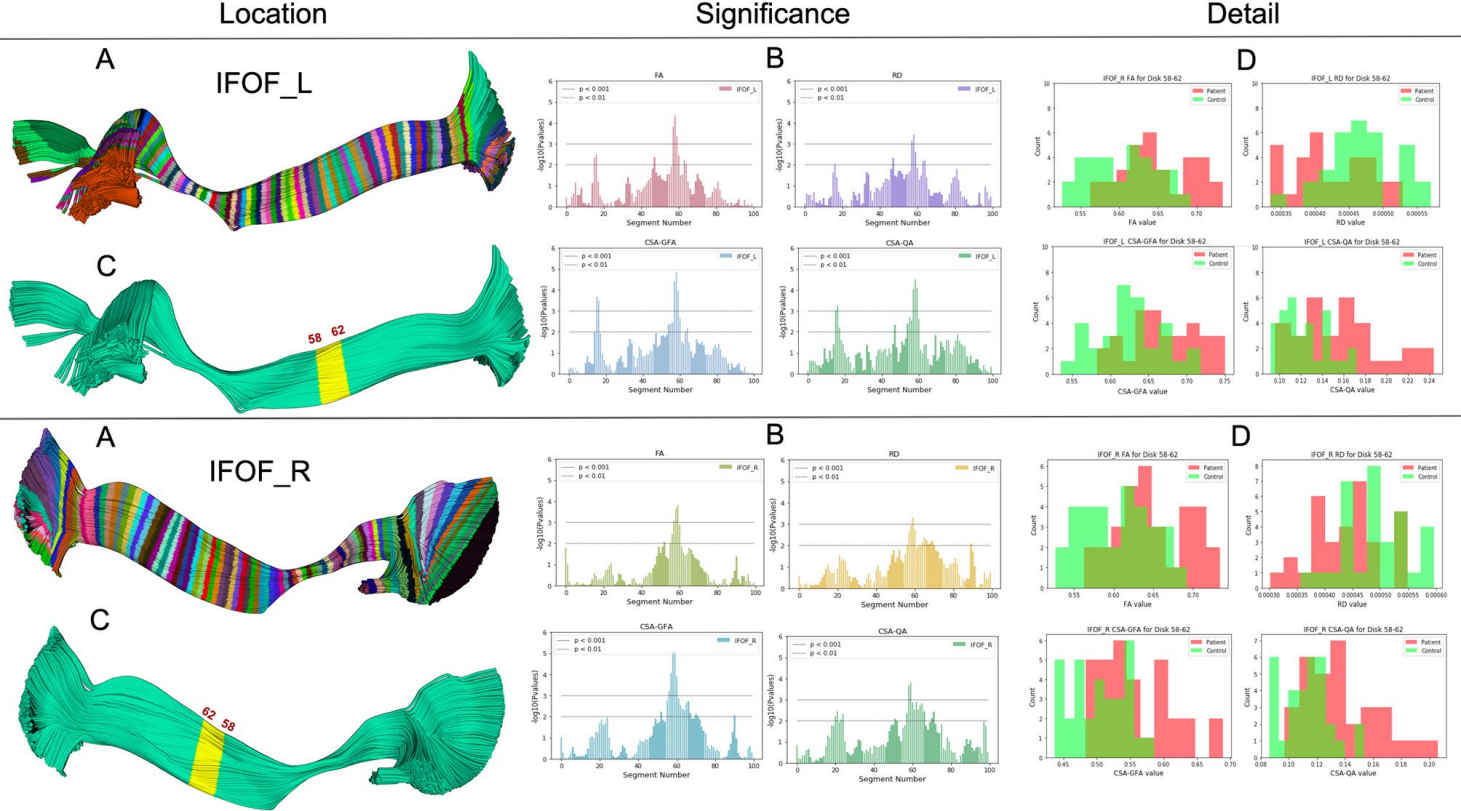
BUAN can identify the significant areas (middle column), visualize and locate them (left column) and then show why this is happening for the population (right column). Here showing the left (top) and right Inferior Fronto-occipital Fasciculus (bottom). Bundles are divided into 100 segments as shown in (A), each segment is given as an input to linear mixed models (LMM) and the output of LMM identifies significant FA, MD, CSA-GFA and CSA-QA group differences at each segment in (B). The highlighted yellow segment in (C) shows the exact location (58-62 segments) on the bundles where the *p* value < 0.001. Locations/segments on bundles are selected by using segments with higher significant differences provided by Linear Mixed Models results. Histograms of mean FA, MD, CSA-GFA, and CSA-QA of control (green) and patient (red) groups at specific significant locations of 58 - 62 segments shown in (D) provide additional explanation of the results. Notice how BUAN not only provides significant results but also shows why these differences were brought up in the populations (see right column).

Fig. 4, depicts BUAN’s bundle profile analysis process. On the top, we have the left Inferior Fronto-occipital Fasciculus (IFOF_L) and on the bottom, we have the right Inferior Fronto-occipital Fasciculus (IFOF_R). For both IFOF_L and IFOF_R, we have (A) bundle divided into 100 segments. (B) shows LMM result plots for IFOF bundles for FA, RD, CSA-GFA, and CSA-QA measures. We can see FA, RD, CSA-GFA, and CSA-QA are significantly different at 58-62 segments on the IFOF bundles for patient and control groups. The highlighted yellow segment in (C) points out exactly where 58-62 segments lie on both left and right IFOF bundles. Locations on the bundles are selected by using segments with higher significant differences (*p* value < 0.001) provided by linear mixed models results. We selected the 58-62 segments on the IFOF bundles of all 64 subjects, 32 controls, and 32 patients and calculated the mean FA, RD, CSA-GFA, and CSA-QA at the highlighted yellow location as shown in (C) per bundle. (D) shows histogram plots for mean FA, MD, CSA-GFA, and CSA-QA of control (green) and patient (red) groups at the location of 58-62 segments on bundles. On the y-axis, we have the number of the subjects, and on the x-axis, we show the FA/RD/CSA-GFA/CSA-QA values. FA, CSA-GFA, and CSA-QA values increase in patients, and RD values decrease in patients as compared to the control group.

### Bundle Shape Similarity

**Figure 5:**
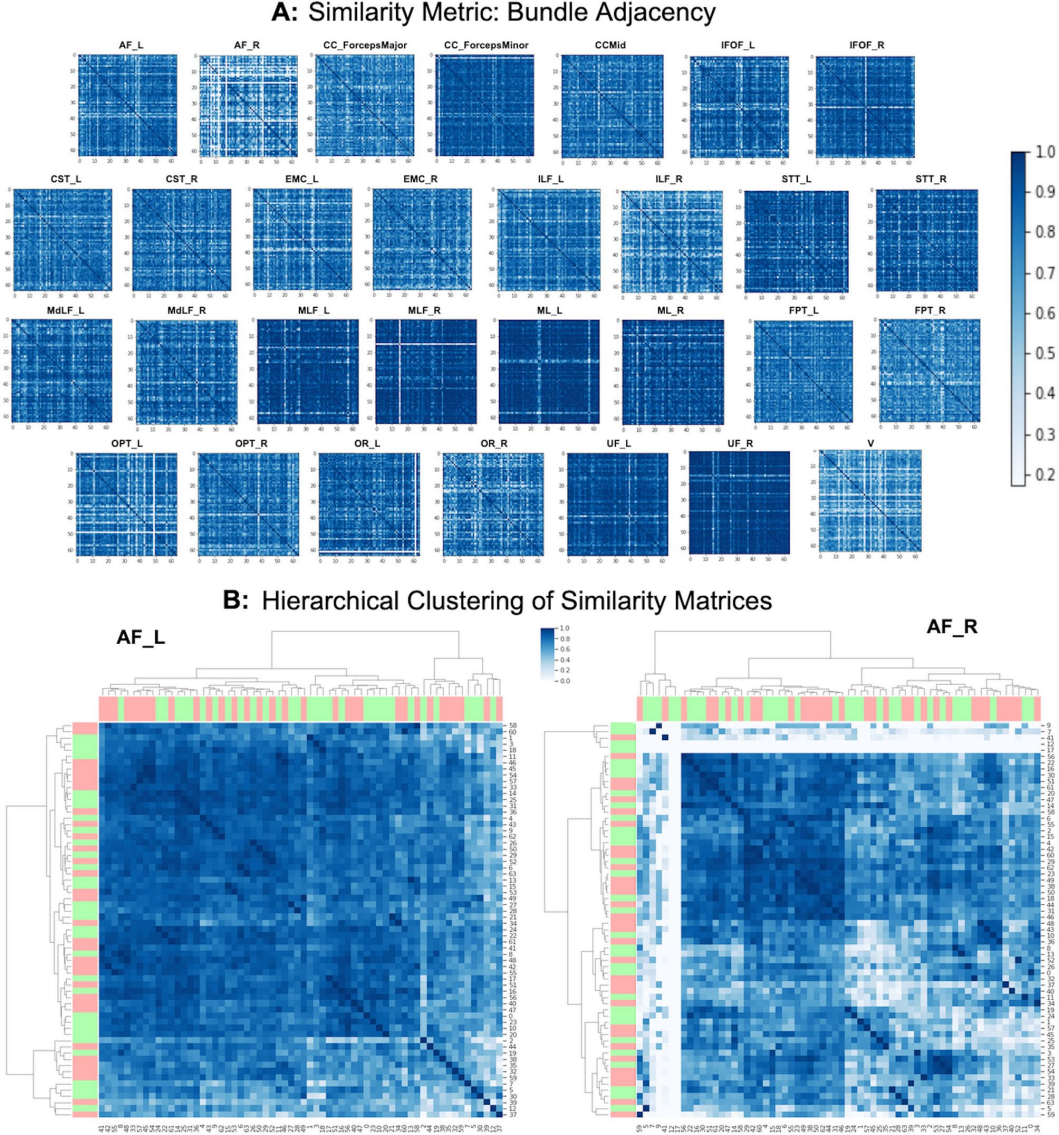
(**A**) Similarity matrices for 30 bundles of 64 subjects. The similarity metric used here is bundle adjacency (BA). The first 32 rows represent healthy controls and the last 32 rows belong to patient data. Higher BA value has a darker blue color which signifies higher shape similarity among bundles. Lower BA value which tends to white color signifies lower shape similarity. The diagonal of every matrix is dark blue (BA score of 1) as it is measured against itself. All similarity scores are calculated with the BA threshold of 5 mm. Notice that we can easily identify from these matrices which bundles are less similar in shape by looking at the color of matrices. (**B**) The hierarchical clustering of similarity matrices of AF are presented. The red bars represent patient observations and green bars represent healthy controls. Notice how the AF_R has overall more white than AF_L. This is expected as the right arcuate fasciculus is not always easily detected in the brains of all individuals.

We performed the shape analysis of the bundles using bundle adjacency (BA) for all the 30 bundles of all 64 subjects. See "Shape analysis using bundle adjacency" section for method explanation. Results generated by bundle adjacency (BA) are used to create a connected bundle graph network that is represented in a compact manner as similarity matrices. One similarity matrix for each bundle. Fig. 5.A, shows the similarity matrices for 30 bundles created using BA for 64 subjects. Each similarity matrix is a $64\times 64$ matrix. The first 32 rows of the matrices (0-31 subjects) belong to the control data, and the last 32 rows of the matrices (32-63 subjects) belong to the patient data. The darker blue color of the similarity matrix means bundles have higher BA scores of shape similarity and blue color converging to white shows the least similarity in the shape among the bundles. BA threshold used is 5mm, which is towards the higher strictness spectrum for calculating the shape similarity among bundles. For some subjects, we cannot extract a specific bundle or the bundle is very thin. In this case, the similarity matrix of the bundle has a white line in the subject’s row and column. Therefore, shape similarity matrices also play the role of quality assurance. Similarity matrices detect outlier subjects who have lower BA scores with other subjects’ bundles. These matrices tell us which bundles are prominent and easily extractable among subjects. Here, we observe, CC_ForcepsMinor, MLF_L, MLF_R, ML_L, ML_R, UF_L, and UF_R to have dark similarity matrices. This implies that shape for all these bundles is highly consistent across the subjects and that sets of bundles are readily extractable in all the subjects. The AF_R, ILF_R, OPT_L, and V have white lines in their similarity matrices. This suggests these four bundles were missing from some subjects and if they were available, they differed in shape overall. Note that the BA threshold being used here is extremely strict in giving similarity scores. Most of the bundles tend to have darker similarity matrices, proving robust and good quality bundle extraction performed by the BUAN framework. In order to find clusters and patterns in the BA similarity matrices, we applied hierarchical clustering on the similarity matrices. Fig. 5.B, shows hierarchical clustering on the similarity matrices of two bundles, AF_L and AF_R respectively. Here, we have added label row and column to show a group of the observation. The observation (subject) belongs to a patient group when the label color is red and it belongs to the control group when the label color is green. Notice that the AF_L bundle has two bigger clusters and the AF_R bundle similarity matrix is divided into five clusters. Hierarchical clustering results of the similarity matrices can be interpreted as clusters that are comprised of subjects who have similar bundle shapes. Results for hierarchical clustering on all 30 bundles can be found in the supplementary materials Appendix A.3 Fig. A7.

### Comparisons with AFQ

**Figure 6:**
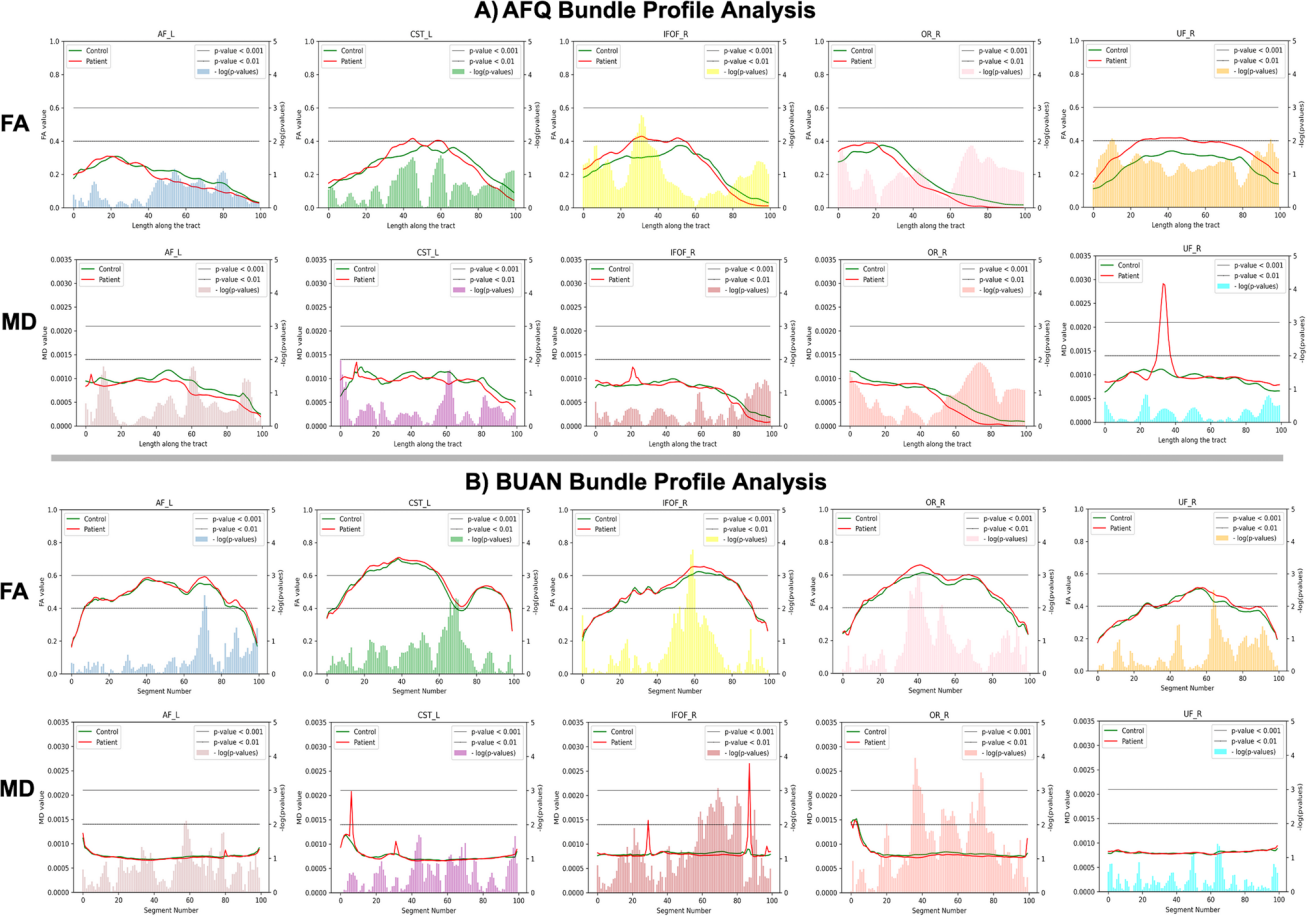
Bundle profile analysis was carried out on PPMI data of 32 control and 32 patient subjects. The figure shows results for five major bundles, AF$\_$L, CST$\_$L, IFOF$\_$R, OR$\_$R, and UF$\_$R. The 2-sample independent t-test was carried out on AFQ bundle profiles of subjects to get significant group differences along the tract. For BUAN, linear mixed models (LMM) was run to get significant group differences along the tract. (**A**) shows AFQ bundle profile results on 5 bundles and (**B**) shows BUAN results on the same data. In both (**A**) and (**B**), the first row has mean bundle plots for fractional anisotropy (FA) and second row has mean bundle plots for mean diffusivity (MD). Each plot has a mean bundle profile for the control group in green and a mean bundle profile for the patient group in red. On the x-axis, we have length along the tract/segment number, on the left y-axis, we have mean anatomical values and the negative logarithm of *p* values on the right y-axis of the plots. In the plots, the horizontal lower line indicates a *p* value < 0.01 and the horizontal upper line indicates a *p* value < 0.001.

The same preprocessing and bundle extraction methods were used in creating Automated Fiber-Tract Quantification (AFQ)^29^ and BUAN bundle profiles. Both AFQ and BUAN bundle profile analyses were run on the PPMI data set comprised of 64 subjects, 32 controls, and 32 patients respectively. Subject bundles and anatomical files used for analysis are the exact same for both bundle profile methods. The AFQ method generates one mean bundle profile per subject, we took an average of 32 patient subjects bundle profiles to create one bundle profile for the patient group and averaged 32 control subjects bundle profiles to create one mean bundle profile to represent the control group. The AFQ does not provide statistical analysis beyond the generation of profiles. To get the areas of significant group differences we ran a 2-sample independent t-test on AFQ bundle profiles along the length of the tract. The AFQ method generates a mean streamline (bundle profile) per subject with 100 equidistant points. For a given bundle, a t-test was run to get group difference significance at each point. In Fig. 6, we present results from two methods using 5 bundles, AF$\_$L, CST$\_$L, IFOF$\_$R, OR$\_$R, and UF$\_$R. In Fig. 6, both A) AFQ and B) BUAN bundle profile analysis results are plotted. In both, the first row has mean bundle FA plots for 5 bundles and mean bundle MD plots in the second row. The control group is shown in the green and the patient group is shown in red. Plots have mean anatomical values on their left y-axis, the x-axis has a length along the tract and the negative logarithm of *p* values is plotted on the right y-axis of plots. In all the plots, the horizontal lower line indicates a *p* value < 0.01 and the horizontal upper line indicates a *p* value < 0.001. In Fig. 6A, for FA, we see group differences in AF_L at 40–90 area of the tract, for CST_L at 30–50, 55–65, and slight difference throughout the rest of the tract, for IFOF_R 1–55 area shows the bigger difference and relatively small difference in rest of the tract, for both OR_R and UF_R there are group differences throughout the tract. In the case of MD, we observe slight changes in groups for all 5 bundles. In the IFOF_R tract, we see a spike in patient group’s mean at 20–25 area along the tract. In UF_R, we see a huge spike in the patient group’s mean at 25-40 areas along the tract. In Fig. 6.B, we consider segments with *p* values in the range of 0.001–0.01 to be significantly different. For FA, we observe significant group changes in AF_L at segment 70, in CST_L at segments 65–70, in IFOF_R at segments 55–60, in OR_R at segments 35–42, and in UF_R at the segment 65. In the case of MD, we observe significant group differences in IFOF_R at segments 58–74, segments 78–82, and segment 90. In OR_R tract at segments 35–42, segments 50–60 and segments 64–76. We do not see significant group differences in AF_L, CST_L, and UF_R tracts.

## Discussion

BUndle ANalytics (BUAN) provides a unique platform for tractometry and connects it to anatomical information for analysis of white matter bundles. BUAN is a fast and robust framework that provides a completely automatic and streamline-based framework for extracting bundles to facilitate the study of the anatomy and shape of those white matter structures. It does not require nonrigid image registration at any step in the pipeline. This is important because image registration is particularly prone to failure when dealing with patient data^47^. None of the methods used in the BUAN framework depend on training data as compared to deep learning methods^15,48^ which need labeled patient data, which can be hard to find. This is important as researchers do not have training data for most clinical cases and BUAN can be directly used for these cases. Deep learning systems^15,48^ can only extract bundles used in training data. Another advantage of BUAN is therefore that new bundles can be found when new atlases or model bundles are provided. BUAN could be extended to be used in the brains of other species. There are no theoretical or other limitations that restrict usage to other species. However, this is a topic for future research and we hope that the community will apply BUAN in many other data sets, including non-human primates and other species. For statistical analysis of bundle profiles, BUAN uses linear mixed models. However, we are planning to include other methods such as functional data analysis^49,50^ and predictive machine learning^37^. Bundle adjacency (BA) is used for shape similarity analysis of the same bundles across subjects. BA can be extended to use any distance metric between streamlines, we are currently using the minimum direct flip (MDF) distance^21^. Other distances can be used too, such as the Hausdorff distance^51^ or MAM distance^52,53^. We can look at shape similarity matrices as a method not only for finding shape similarity among the bundles but also as a metric to check the quality of data. A darker similarity matrix (see Fig. 5) of a given bundle suggests that this bundle is readily extractable across all subjects. BUAN’s end-to-end processing time for 64 subjects was 46.847 hours on a single machine with 32 GB RAM, and one Intel Core i7-7700K CPU with 8 cores. The details about the processing time of each of the BUAN’s steps can be found in supplementary materials Appendix A.5.

### Advantages

Our approach is highly generic, modular, and flexible. It can be adopted for any sort of challenging clinical data sets or any animal data sets. In the case of animal data, the user can provide their own atlas of bundles and can perform bundle analysis using the same pipeline. For adult human data we are already providing an atlas, though users can also use their own atlas. None of the methods described here require a large number of labeled data for training. BUAN uses all the points on the streamlines, as we do not need to reduce the number of points of data points to simplify the statistics. Neither do we need to cut out the extremities of a bundle. It is left to the user to decide at what lengths they want to study a pathway. BUAN does not simplify the final bundle profiles by taking the average of anatomical measure values along the length of the bundle. Most importantly, BUAN does not impose or apply any sort of deformations at any stage of the framework. How deformations affect statistical validity is still an open question^47^ for the field of neuroimaging and therefore, given this uncertainty, we do not deform the data. The sparsity of the tractograms exploited by BUAN is enough to enable high-quality correspondence between the subjects and use the anatomical measures directly in native space. Our methods can also be used for quality control, for example, if multiple model bundles are not being found in the data that means there are very likely issues with acquiring the data.

### Concerns

Our framework results in a large amount of derivative data. In this paper, resulting HDF5 files for 64 subjects (30 bundles) occupied 10 GB. This suggests that the application of this method to 650 subjects would result in the generation of a TeraByte or more of data. These derivative files are important because LMM is applied to them to provide results based on the proper accounting of correlations among the streamlines. However, the analysis can be run per bundle as they are all independent and it is, therefore, permissible to discard the large intermediary files. Therefore, the analysis is scalable to hundreds and thousands of subjects. Bundle extraction method relies on model bundle’s definition of a bundle, it will try to extract a bundle from target data that has similar properties as the model bundle. The more realistic the model bundles the better the extraction. The auto-calibration step helps in extracting the bundle which has shape influence from its own data because the model bundle provided is part of the target tractogram itself. Some bundles, due to their morphology, will be less well summarized by the use of a single centroid. For example, the corticospinal tract exhibits a great deal of fanning in its superior terminations. As future work, it would be worthwhile to attempt to model these more complex white matter tracts with multiple centroids.

We provided the results for the nominal significance level of 0.001 as an illustration in our pipeline. The significance level is a parameter that is chosen by the user. We reported in our manuscript the results at the 0.001 level to illustrate the pipeline. We now provide an additional option in the pipeline, where the user can specify the chosen significance level. We set as default the FWER (family-wise error rate) method. As the tests both within the tract and across tracts are correlated, we use the result on the bounds of correlated tests^54^, and make the following corrections: (1) within-tract adjustment is specified as (the number of points) divided by the (correlation of the test statistics within the tracts); (2) across-tract adjustment is specified as (the number of tracts) divided by the (correlation of the test statistics across the tracts). Thus, for example, if the testing is done at n=100 points within the tract and there are 30 bundles, the corrected significance level is 0.05 divided by $(100/(1-\rho _{within})$ and $(30/(1-\rho _{across}))$; assuming $\rho _{within} = 0.9$ and $\rho _{across} = 0.6$, the corrected significance level is $0.05/(10\times 5) = 0.001$. Proper adjustment for multiple comparisons needs to take into account very high correlations between the adjacent disks. We will address this issue in our future work via a functional data analysis approach treating all the streamlines as functions clustered within a tract.

Using the same MDF metric in bundle extraction method and bundle shape similarity method might overstate the similarity between the bundles. However, the estimated relative difference between any two subject’s bundles will be very similar.

### Connections to previous studies

The inferior fronto-occipital fasciculus is a major long-range tract connecting anatomically distinct cortical brain regions. Previous studies have suggested a role of the IFOF in complex cognitive tasks, including executive function, social cognition, attention, and semantic processing of language, perhaps as different subunits^55–57^. Previous studies in PD have identified changes in the IFOF in PD-dementia (PDD) and Dementia with Lewy Bodies (DLB), as well as associations with executive function, language, attention, and depression in PD patients^58–61^. Previous reports in the PPMI cohort indicated increased FA and reduced diffusivity in the IFOF and other tracts in PD relative to healthy controls, particularly tremor-dominant PD^62,63^. Other tracts showing differences between healthy control (HC) and Parkinson’s disease (PD) patients included the frontopontine tracts, inferior longitudinal fasciculus, occipitopontine tracts, middle longitudinal fasciculus, and uncinate fasciculus. The frontopontinue tracts showed degeneration in progressive supranuclear palsy (PSP), an atypical Parkinsonian syndrome^64^. In addition, alterations in the inferior longitudinal fasciculus were observed in previous reports in PDD, DLB, and PSP, as well as associations with cognition, color vision deficits, and depression in PD^58,59,61,65–68^ The uncinate fasciculus also showed alterations in PD without dementia, PDD, DLB, and PSP, as well as associations with cognition and depressive symptoms^58,59,61,66–69^. The uncinate fasciculus is also involved in olfactory function, which is impaired in patients with PD^70^. In this experiment, we observe a trend towards higher FA, and lower diffusivity in PD patients relative to controls, which may reflect a compensatory response to ongoing pathological changes associated with the disease, such as deposition of alpha-synuclein and degeneration of the substantia nigra and striatum. These findings are similar to previous reports in this sample^62,63^. Increased FA in the motor pathways of PD patients: the bilateral corticospinal tracts, bilateral thalamus-motor cortex tracts, and the right supplementary area-putamen tract have been reported^71^. Increased FA in the precuneus area of patients with PD without mild cognitive impairment has also been reported^72^. Another study has shown an increase in FA and AD in the nigral subareas in PD^73^. However, previous reports have also suggested a faster longitudinal decline in FA and an increase in diffusivity in PD patients relative to controls^63,74^. Patients with early-stage PD had reported higher MD relative to healthy controls^75^. Decreased FA was observed in PD subjects in the frontal lobes, including the supplementary motor area, the pre-supplementary motor area, and the cingulum and no significant differences observed in MD between PD subjects and controls^76^. A test–retest study on PPMI dataset^27^ reported similar results as presented in BUAN. An increased FA and GFA and decreased MD and RD in patients as compared to controls in substantia nigra (SN), the striatum and subthalamic nucleus (STN), pallidum, putamen and thalamus regions of the brain. The same trend was observed in the motor and premotor part of the corpus callosum and corticospinal tract^27^. Future studies with the BUAN framework extending the method to longitudinal DTI data from PPMI are warranted.

### Comparisons

Figure 6 shows plots for only 5 bundles but AFQ and BUAN were run on all 30 bundles. In AFQ, for most bundles average FA values increase in patients as compared to controls and average MD values decrease in patients as compared to controls. Few bundles have areas where FA decreases in patients as shown in Fig. 6A. FA values decrease in AF_L and OR_R bundle. In the case of MD, we see a small spike in MD values of patients in the IFOF_R and a huge spike in patients in the UF_R bundle. This could be caused due to two reasons, (1) anatomical values at the endpoints (extremities) of the tracts change drastically, and (2) one weighted-averaged streamline cannot represent the whole bundle with fanning. When average is taken, one outlier subject can cause the spike in the final plot which happens to be the case in MD plots for IFOF_R and UF_R bundle. In BUAN, significant areas on the tracts with group differences show consistent results. The average FA values increase in patients as compared to healthy controls at all segments with significant group differences. The average MD values decrease in patients as compared to healthy controls at all segments with significant group differences. The reason for the consistent results of average FA and MD values in BUAN is because all points on all streamlines are taken into the account by LMM to find group differences. BUAN utilizes information from all streamlines with different shapes and sizes in the data. In BUAN MD plots, we see a spike in patient mean in CST$\_$L and IFOF$\_$R bundle plots. This could be due to one or two outlier subjects in the patient data. However, LMM does not give higher significance to that area which validates that reported areas of significant group differences in BUAN are not affected by outliers. In the plots shown in Fig. 6, we observe the average FA and MD plots generated by AFQ and BUAN look different. AFQ has a higher mean variation in groups as compared to BUAN results. The reason for these differences could be due to the algorithmic differences between the two methods. These are two different methods with different assumptions about the data. Different lengths of streamlines and how they are weighted can play a role in getting different results from AFQ and BUAN. AFQ discretizes each streamline into N equidistant points and then takes an average of every $i\mathrm{th}$ point of all streamlines to create one weighted averaged streamline per subject where streamlines/points closest to mid of the bundle are weighted more than streamlines far from the mid. As shown in Fig. 8, streamline 2 and streamline 7 end earlier and streamline 5 starts later than the rest of the streamlines (smaller in length than other streamlines). The last point of streamline 2 and streamline 7 will contribute to the average of the 5th point of the final averaged streamline. The last point of both streamline 2 and streamline 7 is far from other streamlines’ last points. The same goes for the first point of streamline 5. This length variation of the streamlines is not taken into account in this experiment by AFQ which can cause a difference in comparisons with BUAN. In BUAN, points are assigned a segment number, and the length of the streamline is taken into account this way. In the case of Fig. 8, the last points of the streamline 2 and streamline 7 are assigned to the closest model centroid point and assigned the 4th segment number. Starting points of the streamline 5 are assigned 2nd segment in a similar fashion. Also, all points on the streamlines are used to take the average per segment. Weights are not given to points while taking the average. In summary, BUAN utilizes all points on the streamlines when taking the average and also takes lengths of streamlines into account. AFQ discretizes points into N equidistant points per streamline and assigns more weight to the streamlines in the middle of the bundle while taking the average. Overall, both AFQ and BUAN report similar WM alterations between patients and controls (increased FA in patients and decreased MD in patients as compared to healthy controls). Further research will be needed in the future to study the impact of different tractometry methods and their assumptions. We hope this framework will help in that direction.

### Conclusion

BUndle ANalytics (BUAN) is a powerful tool for performing analytics of white matter fiber bundles. BUAN provides a completely automatic, end-to-end streamline-based solution that connects bundle recognition, analysis of bundle anatomy, and shape analysis. More importantly, BUAN is the nexus between several successful streamline-based methods^11,21^ of tractography, and other brain imaging modalities. BUAN reports the exact locations of population differences along the length of bundles. Additionally, beyond looking at the profiles of the bundles, BUAN includes individual shape differences of the bundles. For this purpose, we introduced a novel network-based, bundle shape analysis method using bundle adjacency metrics to assess and compare shapes of the same type of bundles, across groups. BUAN is a generic framework that can be applied both in clinical and healthy populations. In theory, BUAN should work with other animal brains with no changes in the code. All methods of BUAN are carefully implemented, thoroughly tested, and made publicly available to the community for use through command-line interfaces, and Python scripts. Scientists can use the BUAN framework to study different types of pathological data and find differences between populations based on tractometry and shape analysis. BUAN is available with DIPY^44^ at dipy.org.

## Methods

### Data

Data used in the preparation of this article were obtained from the Parkinson’s Progression Markers Initiative (PPMI)^46^ database. PPMI is a longitudinal, observational, multi-site study with a goal of identifying and evaluating biomarkers for detecting and monitoring the progression of Parkinson’s disease (PD). Participants were included as PD if they: (1) had two of the following symptoms: resting tremor, bradykinesia, rigidity (must have either resting tremor or bradykinesia) or either asymmetric resting tremor or asymmetric bradykinesia; (2) a diagnosis of Parkinson disease for $\le$ 2 years at screening; (3) Hoehn and Yahr stage I or II; (4) screening dopamine scan (DaTSCANTM or VMAT-2) was consistent with dopamine transporter deficit; (5) not expected to require PD medication within at least 6 months from Baseline; (6) Male or female age $\ge$ 30 years at PD diagnosis. Control subjects were also males or females age $\ge$ 30 years with no first degree relative with idiopathic PD and normal cognition. This dataset contains 179 healthy control subjects and 412 patients recently diagnosed with PD. These details about the data set are available at the PPMI website^46^ and they are also described in this paper^27^. Healthy controls and patients have a mean age of 59 and 61 years respectively. Most of the subjects are Caucasians (93$\%$), 71$\%$ of PD subjects are male, and 57$\%$ of healthy patients are male. PPMI dMRI data was acquired using a standardized protocol used on Siemens TIM Trio and Siemens Verio 3 Tesla MRI machines from 32 different international sites. Diffusion-weighted images were acquired along 64 uniformly distributed directions using a b-value of 1000 $s/mm^2$ and a single b $=$ 0 image. Single-shot echo-planar imaging (EPI) sequence was used (matrix size = $116\times 116$, 2 mm isotropic resolution, TR/TE 900/88 ms, and twofold acceleration). An anatomical T1-weighted 1 $mm^3$ MPRAGE image was also acquired. Each patient underwent two baseline acquisitions and two more one year later. The right and left-onset patients are distributed in proportions of 57$\%$ and 43$\%$. More information on the MRI acquisition and processing can be found online at www.ppmi-info.org. We have processed 64 subjects: 32 controls, and 32 We selected subjects in the age range of 39–61 to eliminate the possibility of any biases introduced by the age differences of the subjects. Each group is comprised of 14 female and 16 male subjects to further eliminate biases introduced by different sex distribution of the subjects in groups. In summary, these were all the valid subjects available in the PPMI dataset that were in the specific age range and also balanced the number of subjects with same sex in both groups.

### Streamline-based Bundle Atlas

In this paper, we used a publicly available streamline-based bundle atlas. Our atlas was a reduced form of the HCP-842 template^77,78^. Original population-based atlas was modified for the BUAN framework. Original atlas comes with 80 bundles. We combined all 80 bundles together to create an atlas of the whole brain tractogram. The whole-brain atlas tractogram and 80 bundles were moved to MNI 152 space (ICBM 2009a). Lastly, 30 bundles of interest were selected out of 80 bundles to be used in the analysis. We excluded bundles from the atlas that did not exist in the PPMI data. Many of the bundles in the original atlas were cranial nerves (pathways outside of the brain) that do not exist in this data. Our neuro-anatomist inspected the atlas and removed any bundles or streamlines with unrealistic shapes that did not confirm the anatomy as reported in^5,12^. The final atlas can be downloaded from DIPY^44^ using the $\text {dipy}\_\text {fetch}$ command. The atlas contains a whole-brain tractogram and the corresponding subset of the 30 bundles. Names of the bundles can be found in supplementary materials Appendix A.1 Fig. A1.

### Data preparation

The local principal component analysis (LPCA) noise reduction method^79^ was used for the diffusion MR images. For brain tissue extraction, the median Otsu algorithm^80^ was used. The distortions induced by eddy currents and motion were corrected by registering the diffusion-weighted volumes to the b0 volume. An affine transformation was computed to register b0 volume and non-b0 3D volumes by maximization of normalized mutual information. The optimization strategy used in DIPY is similar to that implemented in ANTs^81^. The B-matrix (b-vectors) were rotated to preserve the correct orientational information as described in this paper^82^. After the diffusion data is denoised, DTI measures, fractional anisotropy (FA), mean diffusivity (MD), radial diffusivity (RD), and axial diffusivity (AD)^35,36^ were extracted from each subject’s data. A spherical harmonics model based on QBall-Constant Solid Angle were fitted to get orientation density functions (ODFs) and extracted generalized fractional anisotropy (CSA-GFA)^38,39^, and quantitative anisotropy (CSA-QA)^40^. For generating whole-brain tractograms, a constrained spherical deconvolution (CSD) model was used to get directional information from dMRI data^83^, from which a simplified peaks representation was extracted. The obtained peaks were then used as the input to a local tracking algorithm. We performed deterministic tracking^84,85^ using EuDX^52^. EuDX tracking algorithm was initialized with following parameters, tracking starts from voxels where fractional anisotropy (FA) $>0.3$, number of seeds per voxel $=$ 15, step size $=$ 0.5, angular threshold $=$ 60 degrees, and stopping tracking if FA value drops below 0.1. Each generated tractogram comprised of 5–10 million streamlines. DIPY’s^44^ implementation of methods was used in all preprocessing steps.

### Bundle recognition

Tractography algorithms generate potentially unmanageably large data sets with millions of streamlines. It is a logistically challenging task to visually and/or computationally inspect any given individual streamline for aberrant or otherwise distinctive traits looking at the whole tractogram. Thus, our method greatly simplifies this process by extracting specific subsets of related streamlines from the otherwise intractable mass of streamlines. This process of extracting specific groups of streamlines with similar anatomical characteristics from whole-brain tractograms is called automated bundle extraction or bundle recognition. Bundle recognition^11^ goes a step further from automated bundle extraction as it requires no training as it learns from single examples of bundles. In our approach, in order to extract white matter fiber tracts from whole-brain tractogram, we first register an input target tractogram (A.a) to a model tractogram (A.b) using Streamline-based Linear Registration (SLR)^45^ as shown in Fig. 1. After we have transformed the target tractogram from native space to common space, we begin extracting bundles. Whole-brain tracrograms contain a large number of false positives for example, unrealistically small or extremely long streamlines. We pre-processed the tractogram by removing streamlines smaller than 30 mm. The input to RecoBundles (RB) is the registered target tractogram (A.c) and a model bundle (A.d)^11^. The model bundle is used as a reference bundle to find corresponding streamlines in the target tractogram. The model bundle is part of the atlas used for registering the target tractogram to common space. Both target tractogram and model bundle are in common space. The first step in RecoBundles is to reduce the search space and find neighboring areas for the model bundle in the target tractogram. This is achieved by a process called far pruning. We exclude the streamlines from our search space whose MDF^21^ distance with model bundle streamlines is greater than the reduction threshold. The default value or reduction threshold is set to 15 mm. Now, the search space consists of only potential bundle streamlines, we call these streamlines neighbor streamlines of the model bundle in the target tractogram. In the next step, RecoBundles applies local registration of neighbor streamlines to model bundle streamlines using SLR^45^. After local registration of streamlines, local pruning of neighbor streamlines is performed in a similar manner as far pruning. Neighbor streamlines whose MDF distance with model bundle streamlines is greater than the pruning threshold are discarded. The default pruning threshold is set to 8 mm.

### Auto-calibration

Human brain pathways come in all sorts of shapes and sizes. While larger bundles are easier to locate and extract, it becomes difficult to reliably extract smaller and hard to find pathways in large tractograms. To resolve this problem and eliminate the need for changing parameters to deal with short bundles, we added one more step in RecoBundles (RB)^11^ algorithm. We call this new step, auto-calibration. As shown in Fig. 1A. during the auto-calibration step, the final extracted bundle output of standard RB (e) becomes our new model bundle. RB is run again on the same target tractogram but this time the model bundle is not part of an atlas but part of the target tractogram itself. Since the new model bundle is part of the target tractogram, it eliminates the need for local streamline-based registration with the model bundle. All steps of RB are performed again in the same order as described in "Bundle recognition" section except the local registration of neighbor streamlines with the model bundle. The default auto-calibration reduction threshold is set to 12 mm and the auto-calibration pruning threshold is set to 6 mm. This additional step allows us to: (a) use the same parameters for short and long bundles, (b) produce more dense bundles than before, and (c) reduce any issues because of shape differences between the initial model bundle and the final target bundle. We found auto-calibration to be especially useful when dealing with noisy data or when extracting small bundles like the uncinate fasciculus (UF) from the whole brain tractograms. All bundles are extracted using the same default parameters. At the end of the bundle extraction process, we assess the quality of bundle extraction by using two cost functions, bundle adjacency (BA) and bundle-based minimum distance (BMD)^45^. Bundle adjacency of the extracted bundle is calculated with the model bundle to report the shape similarity score between two bundles. BA evaluates the shape similarity between the two bundles. The bundle adjacency method is explained thoroughly in "Shape analysis using bundle adjacency" section. BMD is used for calculating a distance between two bundles. BMD function is explained in detail with equations in Appendix A.6.2 of the supplementary materials.

### Assignment maps

For tractometric studies along the length of the tracts, we are combining information from extracted bundles and anatomy. To perform analysis on extracted bundles, we create assignment maps by dividing the bundles into segments using the model bundle centroids along their lengths in common space (model tractogram space). We cluster our model bundle using QuickBundles^21^ resulting in a cluster centroid (b) with 100 points per centroid as shown in Fig. 1B. To divide a given bundle into segments, we calculate Euclidean distances between every point on every streamline of the bundle (B.a) and all model bundle centroids (B.b) and assign the considered point to the nearest segment centroid similar to^27^. However, we do not resample our streamlines to have a discrete number of points or change the distribution of points. Our approach of creating segments does not require a streamline to have a specific number of points. We use all the points of the streamlines, and assign them to the closest points of the centroid on the model bundle to create assignment maps/segments (B.c). Assignments are created in a common space (model atlas), which ensures that the segment index corresponds to the same centroid across all individuals. Fig. 1B, presents the visual process of creating assignment maps.

The advantage of creating these assignment maps and diving bundles into segments is that we are able to analyze specific areas of a bundle. White matter tracts have distinct shapes and their shapes vary across the length of the tract. For example, in the case of the arcuate fasciculus, we see a larger spread of streamlines at both ends of the bundle. Anatomical measures such as FA also change throughout the bundle. It makes sense to look at a specific segment that has a consistent shape and anatomical measure values.

Information such as the positions of the points of each streamline, the index of the streamlines in the bundle, the segment number, the subject ID, group ID, and anatomical measure values like FA, MD, RD, AD, CSA-GFA, and CSA-QA were saved in HDF5 (.hd5) files. Those files were used as input to statistical models. We used linear mixed models (LMM) for group comparisons based on a given metric (e.g. FA) over a specific tract and specific tract location (segment).

### Linear mixed models

We used linear mixed-effects models (LMMs)^41–43^ to study how anatomical measures differ between patient and control groups along the length of white matter bundles. We applied LMMs separately for each segment of a given white matter bundle. Our main interest was in differentiating between the groups: patients vs. controls. We achieved that by including the group effect as a fixed effect in the regression model describing the outcomes’ (FA, AD, RD, MD, CSA-GFA, and CSA-QA) differences between the groups. To account for the correlations among the observations from different streamlines collected from the same individual and tract, we included random subject-tract effects. See supplementary materials, Appendix A.4 for a detailed mathematical explanation.

### Shape analysis using bundle adjacency

We introduce, a new network-based approach to perform shape analysis between bundles. For getting the resemblance between two bundles, we use the bundle adjacency (BA) metric first introduced in^21^. We use bundle adjacency (BA) to calculate the shape similarity between the same type of bundles across subjects and groups. The higher the BA value, the higher the similarity between the shapes of the two bundles. We created a network graph and similarity matrix from BA values for the same bundle-type across subjects. Bundle adjacency (BA) is calculated between two bundles. BA uses a minimum direct flip (MDF) distance^21^ to get the distance between two streamlines. The minimum average direct-flip distance^21^ is a symmetric distance function that deals with the streamline bi-directionality problem. It calculates both direct and flipped distances between two streamlines which have the same number of points and takes a minimum of both. For equations and more details on MDF distance please see Appendix A.6.1 in the supplementary materials. Let, B1 and B2 be two sets of streamlines (bundles), and let $\theta > 0$ be a selected adjacency threshold. We will say that $b1 \in B1$ is adjacent to B2 if there is at least one streamline $b2 \in B2$ with $MDF(b1, b2)\le \theta$. We define the coverage of B1 by B2 as the fraction of B1 that is adjacent to B2. Coverage ranges between 0 (when all streamlines in B1 are too far from B2) and 1 (when every streamline in B1 is adjacent B2). In order to compare two bundles of possibly different data sets, we define the symmetric measure bundle adjacency (BA). BA is the average of the coverage of B2 by B1 and the coverage of B1 by B2:

$$\begin{aligned} BA(B1, B2) = 0.5 (coverage(B1, B2) + coverage(B2, B1)) \end{aligned}$$

BA ranges between 0 and 1. BA score is 0 when no streamlines of B1 or B2 have neighbors in the other set, and 1 when they all do.

**Figure 7:**
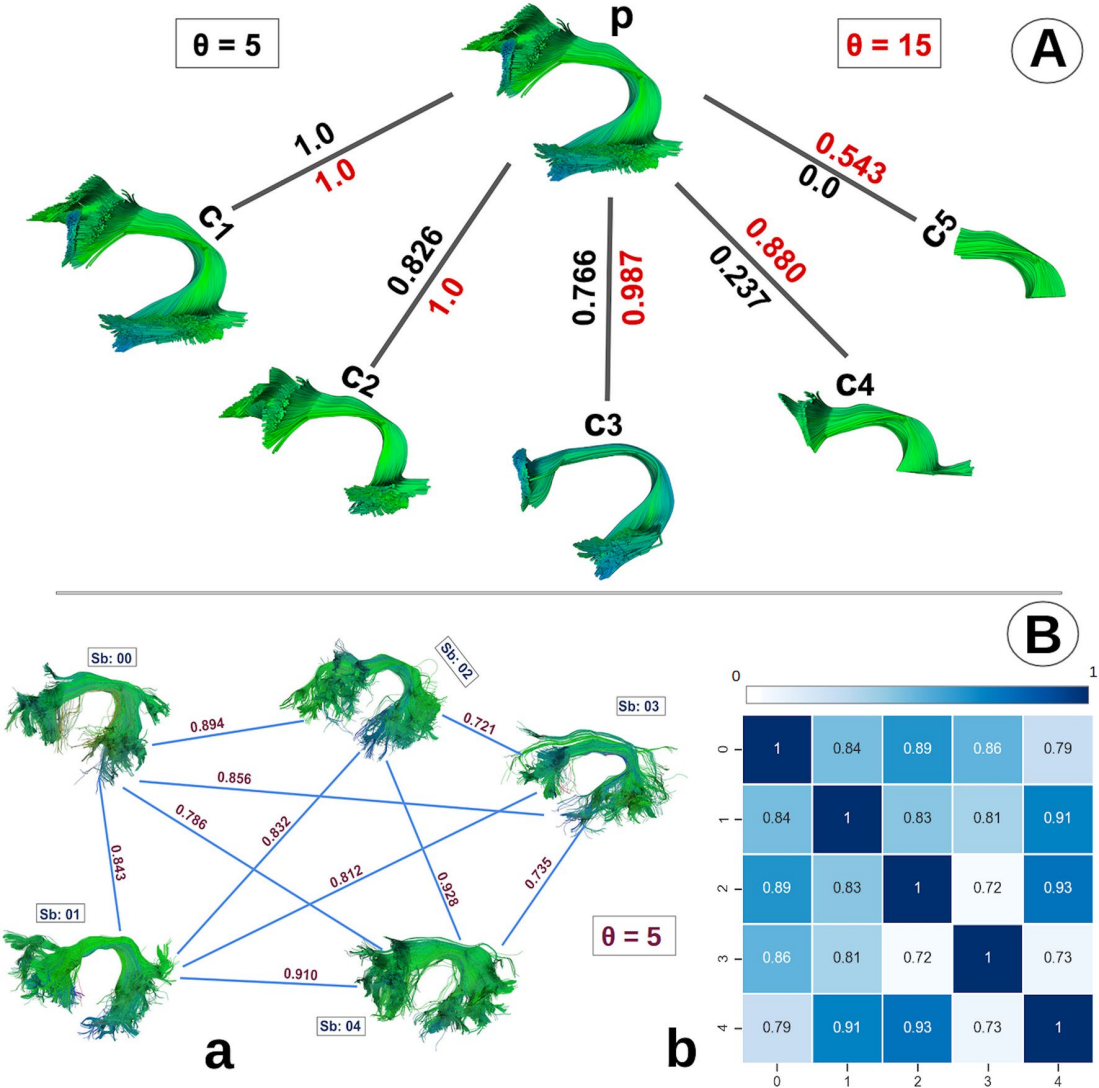
Bundle-based shape analysis. (**A**) shows shape similarity among a parent bundle *p* with 5 children bundles in a tree structure. We calculate shape similarity using bundle adjacency (BA) between parent *p* (AF_L) and children *c*1, *c*2, *c*3, *c*4,  and *c*5 which are curated by eliminating parts of the same parent bundle *p*. The *c*1 bundle is an exact replica of the *p* bundle and it has a perfect BA score of 1. BA score changes as we eliminate larger parts of the bundle in child bundles. For *c*5, we see the least similarity with the BA score of 0 in the case of $\theta =5$. We can manage how strict we want our similarity/shape analysis by adjusting the threshold $\theta$ accordingly. The red threshold of 15 mm is lenient while the black threshold of 5 mm is more strict as shown in (**A**). (**B**) we can illustrate our shape analysis overview as a fully connected graph with similarity (BA) scores on the edges and the bundles as nodes as shown in (a). We can also visualize it as a similarity matrix (b) where dark blue color means higher shape similarity and white color indicates lower shape similarity.

Figure 7, shows the bundle network graph and similarity matrix. Figure 7A, illustrates the concept of bundle shape analyses using the bundle adjacency metric. We can understand this by looking at the diagram as a tree where parent node *p*, the left arcuate fasciculus (AF_L) bundle, for example, has 5 child nodes, *c*1, *c*2, *c*3, *c*4,  and *c*5. Here, the tree nodes are the bundles. These children bundles are created by selecting a subset of the parent bundle streamlines *p*. The *c*1 bundle is an exact replica of *p*, *c*2 bundle is missing parts of the *p* bundle. We created all 5 child bundles in similar fashion by removing significant parts of the original bundle *p* as we move from the left to the right. At the end, the c5 bundle contains only the middle part of the original *p* bundle.

The Bundle Adjacency method requires two bundles and a threshold $\theta$. Where, $\theta$ determines the degree of stringency of shape comparison between two bundles. The higher the value assigned to $\theta$, the easier it is for two bundles to obtain a high BA score, while the lower the value assigned to $\theta$, the lower the resultant BA score.

In Fig. 7, the branches of the tree represent the BA score between two bundles. The color of the BA score represents the threshold value, $\theta$. The black-colored BA score label corresponds to $\theta = 5$ mm used while calculating the BA score. The red-colored BA score label corresponds to $\theta = 15$ mm used while calculating the BA score. We show in the Fig. 7, how one can adjust the bundle adjacency method to produce results for shape similarity based on a threshold $\theta$ which can be seen as a parameter for leniency. With leniency, we mean how loosely we want our method to classify shape similarity between bundles. For example, if we want our bundles to be extremely close in shape by having a rigidly similar length and width, we set the threshold to be a smaller number (5 mm in this example). Which gives a BA score of 1 if the bundle is an exact replica of itself, and it gives a BA score of 0 when no similarity whatsoever which is the case with *c*5.

In the case where $\theta = 15mm$, we can see perfect BA score between *c*1 and *p* which is also the case when $\theta = 5mm$. But for the *c*5 bundle, we get a BA score of 0.543. Although the *c*5 is quite different in shape, it still has some part of the parent bundle *p* (the middle part). The usage or selection of threshold depends on user-specific needs. If we strictly want two bundles to have the same length and width we can use smaller threshold value and if we want to see if a given bundle has sub parts of other bundles or it has some relation with other bundles we can use higher threshold value. BA requires two bundles to be in the same coordinate space to give interpretable results.

### Bundle networks

Results generated by the bundle adjacency function can also be represented as a fully connected network graph of bundles as shown in Fig. 7B. We created a fully connected graph with a similarity (BA) score on the edges connecting two vertices (bundles) shown in (B.a). Here, we have created a fully connected left arcuate fasciculus (AF_L) bundle graph of 5 subjects. In this paper, for each of the 30 different bundles, we created a fully connected network graph of bundles from 64 subjects. Where every subject’s bundle is connected to every other subject’s bundle. The weight on the edges connecting two bundles signifies the strength of shape similarity relationship between two bundles. The higher the weight, the higher the shape similarity.

When there are many subjects (vertices), it becomes difficult to see shape similarity visually only from a connected network graph. For this purpose, we interpret results in a compact way as a similarity matrix (B.b), where darker blue color means higher similarity and lighter blue color indicates less shape similarity among bundles. In the diagonal, we have all 1s as BA is calculated between a bundle and itself. BA can be used as a quality assurance measure, it can also be used to detect outliers in the dataset. When a subject’s bundle is comparatively different than other subject’s bundles, BA gives it a lower score and we can detect that subject’s bundle as an outlier from the similarity matrix.

Using graph-theoretic analysis we can study differences between similar types of bundles. The hierarchical clustering of the similarity matrix is a useful technique to find clusters in the data. We can also represent our similarity matrix as a Voronoi diagram. In this paper, we are applying Ward’s hierarchical clustering^86^ on similarity matrices to find clusters of subjects with similar bundle shapes.

### Method comparison

Automated Fiber-Tract Quantification (AFQ)^29^ has gained popularity in the past couple of years. AFQ is an open-source and freely available software that provides methods for extracting bundles and quantifying diffusion measurements along the length of the white matter fiber tracts (bundle-profiles) that can be used for group comparisons. Originally, AFQ provided region-of-interest (ROI)-based fiber tract extraction method for 18 white matter tracts. AFQ now enables users to select from ROI based bundle extraction or RecoBundles^11^ bundle extraction. After bundles are extracted from the data, bundle profiles are generated based on anatomical measures. For every subject, a mean bundle profile is generated with mean values of any given anatomical measure along the length of the tract. The mean bundle profile is generated by resampling each streamline in the bundle into 100 equidistant points and calculating the mean location of each point. Mean anatomical measurements (eg. FA) are calculated at each point by taking a weighted average of the FA measurements of each individual streamline at that point. Weights are calculated based on the Mahalanobis distance of each streamline point from the core streamline. The final output of AFQ per subject is a mean bundle profile with 100 equidistant points and anatomical measurement values associated with each point along the tract.

**Figure 8:**
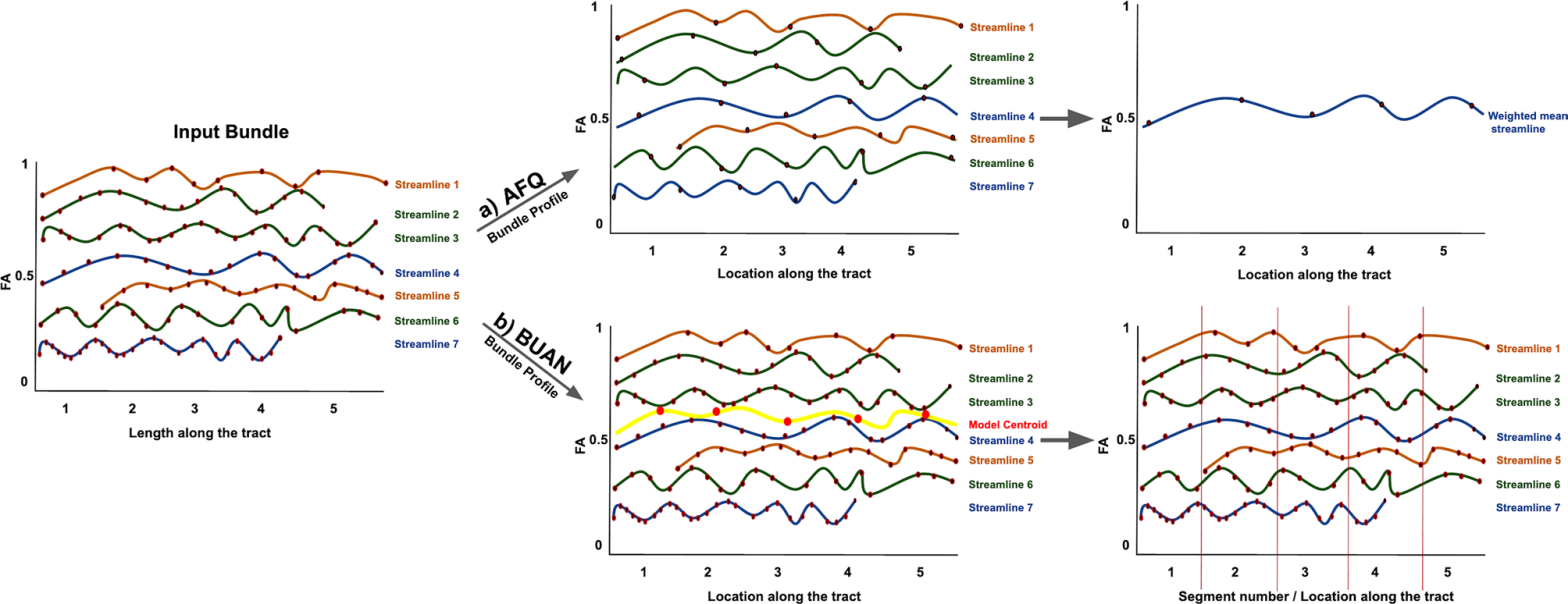
Input bundle is given to (a) AFQ and (b) BUAN to generate bundle profiles. (a) Shows AFQ bundle profile representation for one subject. Each streamline is resampled to have 5 points and then all streamlines are averaged together to create one FA bundle profile. (b) Shows BUAN bundle profile representation of the same subject. The input bundle is divided into 5 segments. Each point on the streamline is assigned a segment number based on the closest centroid on the model centroid streamline (yellow).

Both AFQ and BUAN generate bundle profiles with anatomical measures associated with it along the length of the tract. Figure 8 illustrates the representation of AFQ and BUAN bundle profiles for one subject. This is not real data but simulated for the sake of explanation. Here, the AFQ bundle profile is created by resampling each streamline in the input bundle into 5 equidistant points and then taking a weighted average of all points along the length of the tract. One weighted mean streamline represents the whole bundle. Whereas, in the BUAN bundle profile, streamlines are not discretized into 5 points. The input bundle is divided into 5 segments. Each point on a streamline is given a segment number (label) based on which centroid is it closest to on the model centroid streamline. In the BUAN bundle profiles, all points on all streamlines are kept and are not represented as one simplified averaged streamline.

Note that in the AFQ bundle profile, the last few points on the streamline 2 and streamline 7 are discretized and represented by one 5th point. The 5th point of streamline 2 and streamline 7 is far from the rest of the streamlines’ 5th point. AFQ will take a weighted average of all streamlines’ 5th points to create averaged 5th point of final AFQ bundle profile streamline. Whereas in the BUAN bundle profile, the last points of streamline 2 and streamline 7 are closest to the 4th model centroid point and therefore are assigned to segment number 4. AFQ does not provide any statistical analysis method that can be applied to bundle profiles. User has to visually look at mean bundle plots of subjects and find differences in plots manually. On the other hand, BUAN provides a sophisticated method to automatically find group differences for a given anatomical measure along the length of the tract. BUAN uses linear mixed models for finding group differences. BUAN provides a visualization tool that highlights the exact area on the bundle which is significantly different in two groups. AFQ does not provide any bundle shape analysis method. BUAN provides a novel graph-based bundle shape similarity method explained in "Shape analysis using bundle adjacency" section that can also be used for quality assurance.

## Supplementary information

Supplementary Information 1.

## Data Availability

The Parkinson’s Progression Markers Initiative (PPMI)^46^ is publicly available database https://www.ppmi-info.org. A subset of the datasets generated and analyzed during the current study is released as examples with the pipeline, and complete data set processed is also available. PPMI data derivatives generated during this study can be found at https://doi.org/10.35092/yhjc.12033390. It contains PPMI data derivatives for 64 subjects used in the paper, each subject contains 30 white matter bundles in common space (MNI space), same 30 bundles in subject’s original space, and files containing anatomical information (FA, MD, RD, AD, CSA Peaks). Streamline-based bundle atlas used in this paper can be downloaded from DIPY^44^ using the $\text {dipy}\_\text {fetch}$ command or from figshare. The atlas contains a whole brain tractogram and the corresponding subset of the 30 bundles.

## Code availability

Bundle extraction tutorial can be found here. To reproduce the results on PPMI data, please follow the BUAN tutorial in DIPY. https://github.com/dipy/dipy/blob/master/doc/interfaces/buan_flow.rst. Code for Bundle Analytics framework is publicly available in GitHub and can be found here https://github.com/dipy/dipy.
