# Supplementary material for: Bundle analytics, a computational framework for investigating the shapes and profiles of brain pathways across populations
Source: Sci Rep. 2020 Oct 13;10:17149. doi: 10.1038/s41598-020-74054-4 (PMC7555507; doi:10.1038/s41598-020-74054-4)
Supplement: Supplementary file 1 — Supplementary Information 1. [file 41598_2020_74054_MOESM1_ESM.pdf]

Bundle analytics, a computational framework for investigating the shapes and profiles of brain pathways across populations (Supplement)

Bramsh Qamar Chandio[a 1], Shannon Leigh Risacher[g], Franco Pestilli[c], Daniel Bullock[d], Fang-Cheng Yeh[e], Serge Koudoro[a], Ariel Rokem[f], Jaroslaw Harezlak[b], Eleftherios Garyfallidis[a]

[a] *Department of Intelligent Systems Engineering, Luddy School of Informatics, Computing and Engineering, Indiana University Bloomington, USA.* [b] *School of Public Health, Indiana University Bloomington, USA.* [c] *Department of Psychology, The University of Texas, Austin, Texas, USA.* [d] *Department of Psychological and Brain Sciences, Indiana University Bloomington, USA.* [e] *Department of Neurological Surgery, University of Pittsburgh, USA.* [f] *Department of Psychology and eScience Institute, University of Washington, USA.* [g] *Indiana University School of Medicine, USA.*

[1] *Corresponding author: bqchandi@iu.edu*

Appendix A Appendix

Appendix A.1 Bundle Names

White Matter Tracts Used in the Paper

Projection Pathways

Abbreviation	Full Name
CST_L/R	Corticospinal Tract - Left / Right
ORL_/R	Optic Radiation - Left / Right
FPT_L/R	Frontopontine Tract - Left / Right
OPT_L/R	Occipito Pontine Tract - Left / Right

Commissural Pathways

Abbreviation	Full Name
CCMid	Corpus Callosum Middle
CCForcepsMajor	Corpus Callosum Major
CCForcepsMinor	Corpus Callosum Minor

Cerebellum Pathways

Abbreviation	Full Name
V	Verma

Association Pathways

Abbreviation	Full Name
AF_L/R	Arcuate Fasciculus - Left / Right
EMC_L/R	Extreme Capsule - Left / Right
IFOF_L/R	Inferior Fronto-occipital Fasciculus - Left / Right
ILF_L/R	Inferior Longitudinal Fasciculus- Left / Right
MdLF_L/R	Middle Longitudinal Fasciculus - Left / Right
UF_L/R	Uncinate Fasciculus - Left / Right

Brainstem Pathways

Abbreviation	Full Name
ML_L/R	Medial Lemniscus - Left / Right
MLF_L/R	Medial Longitudinal fasciculus - Left / Right
STT_L/R	Spinothalamic Tract - Left / Right

Figure A1. Abbreviations and full names of the atlas bundles used in the paper.

Fig. A1, lists the abbreviations of 30 bundles used in the paper and their full names. These 30 bundles are selected from the streamlines-based bundle atlas[1] and are used for bundles extraction using RecoBundles[2]. From each subject we extracted these 30 bundles. Atlas bundles are available in DIPY[3].

Appendix A.2 LMM Result Plots

Linear mixed models (LMM) result plots for radial diffusivity (RD), axial diffusivity (AD), mean diffusivity (MD),[4,5], Constant Solid Angle - generalized fractional anisotropy (CSA-GFA)[6,7], and Constant Solid Angle - quantitative anisotropy (CSA-QA)[8].

In the figure for each measure, most plots show left and right bundles of the same type except V and CCMid. The plot at first row, the second column shows simultaneously Minor and Major Forceps of CC. The rest of the plots show the left and right parts of the same type of bundle e.g. AF left(AF_L) vs AF right (AF_R). Notice that the IFOF bundles (left and right) are truly significantly different around segment 60 for all the measures.

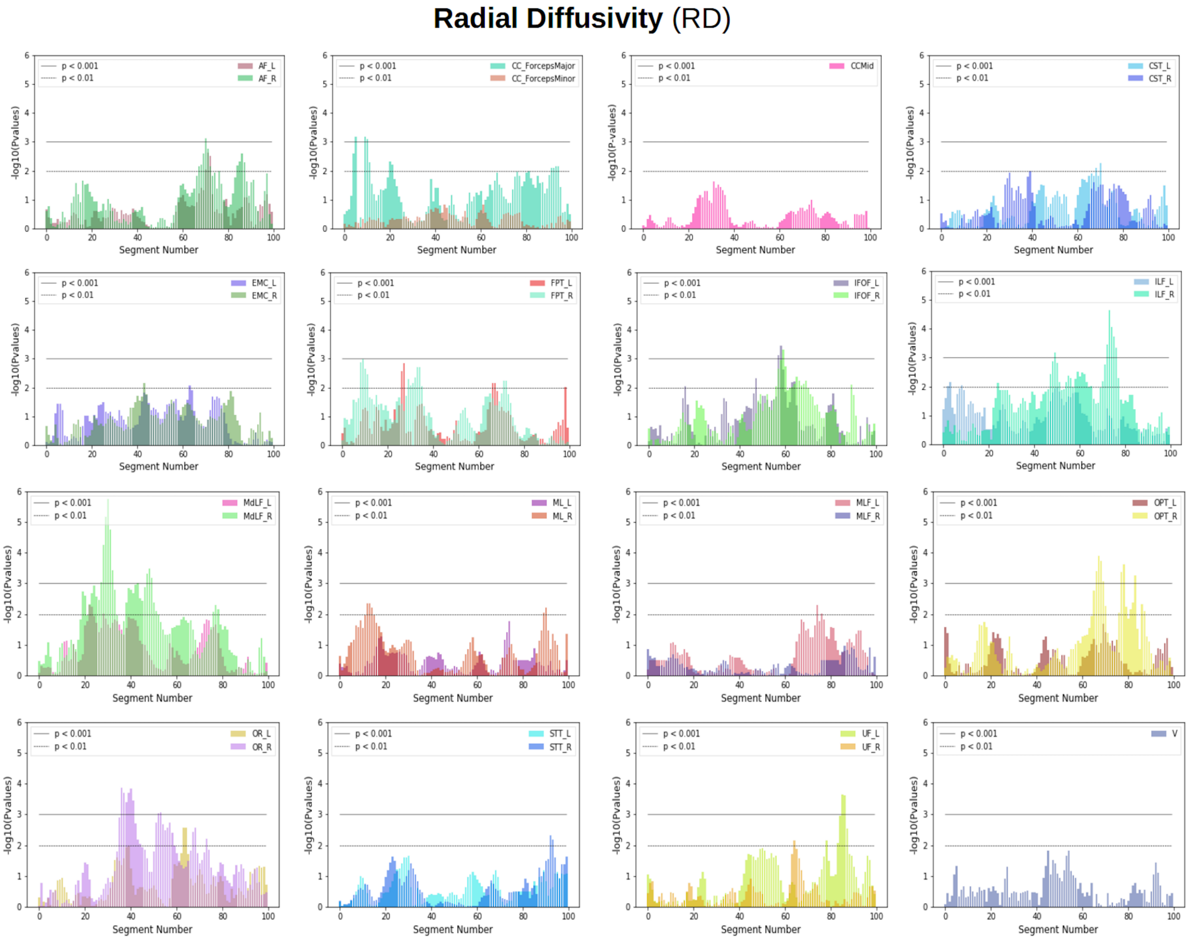

Figure A2. Plots summarizing population differences for radial diffusivity (RD) using linear mixed models for 30 bundles along their length. On the x-axis, we have segment numbers and on the y-axis, we have negative logarithms of p-values. Here, the horizontal lower line indicates a p-value < 0.01 and the horizontal upper line indicates a p-value < 0.001. The p-value at a specific segment implies how much significant RD group differences are there between patients and healthy controls for that particular bundle.

Fig. A2, shows LMM result plots of RD measure. We found significant group differences in AF_R, CC_ForcepsMajor, FPT_R, IFOF_L, IFOF_R, ILF_R, MdLF_R, OPT_R, OR_R, and UF_L bundles.

Axial Diffusivity (AD)

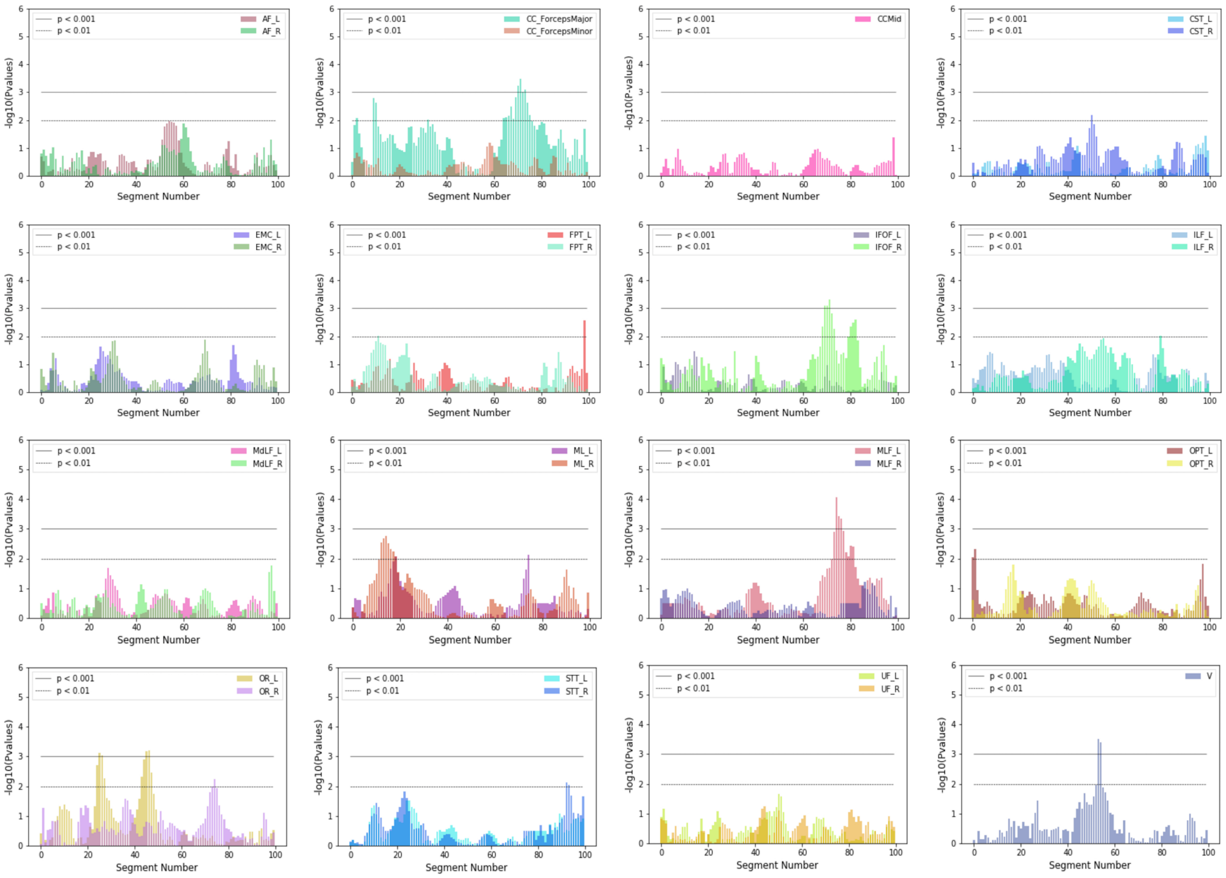

Figure A3. Plots summarizing population differences for axial diffusivity (AD) using linear mixed models for 30 bundles along their length. On the x-axis, we have segment numbers and on the y-axis, we have negative logarithms of p-values. Here, the horizontal lower line indicates a p-value < 0.01 and the horizontal upper line indicates a p-value < 0.001. The p-value at a specific segment implies how much significant AD group differences are there between patients and healthy controls for that particular bundle.

Fig. A3, shows LMM result plots of AD measure. We found significant group differences CC_ForcepsMajor, IFOF_R, ML_R, MLF_L, OR_L, and V bundles.

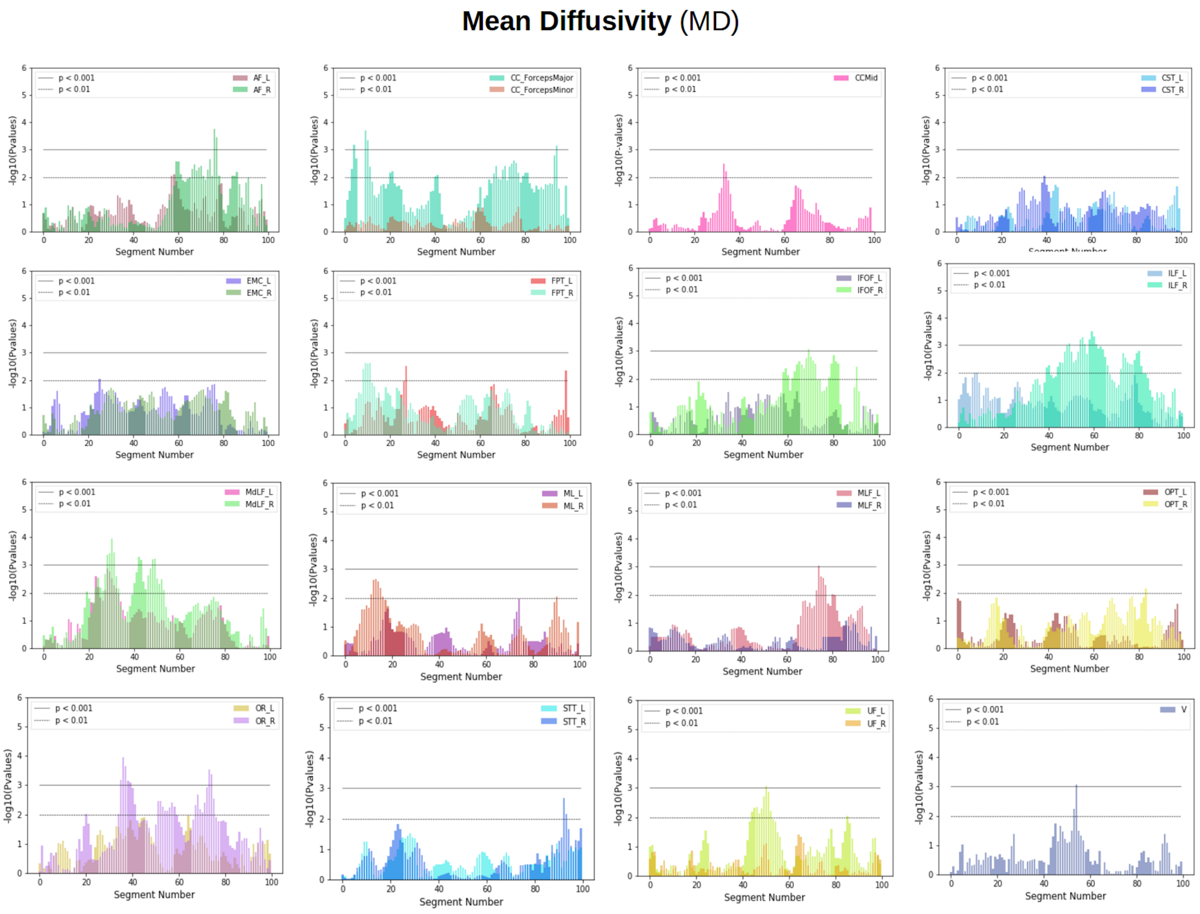

Figure A4. Plots summarizing population differences for mean diffusivity (MD) using linear mixed models for 30 bundles along their length. On the x-axis, we have segment numbers and on the y-axis, we have negative logarithms of p-values. Here, the horizontal lower line indicates a p-value < 0.01 and the horizontal upper line indicates a p-value < 0.001. The p-value at a specific segment implies how much significant MD group differences are there between patients and healthy controls for that particular bundle.

Fig. A4, shows LMM result plots of MD measure. We found significant group differences AF_R, CC_ForcepsMajor, FPT_R, IFOF_R, ILF_R, MdLF_L, MdLF_R, ML_R, ML_L, OR_R, and UF_L bundles.

Constrained Spherical Deconvolution - Generalized fractional anisotropy (CSD - GFA)

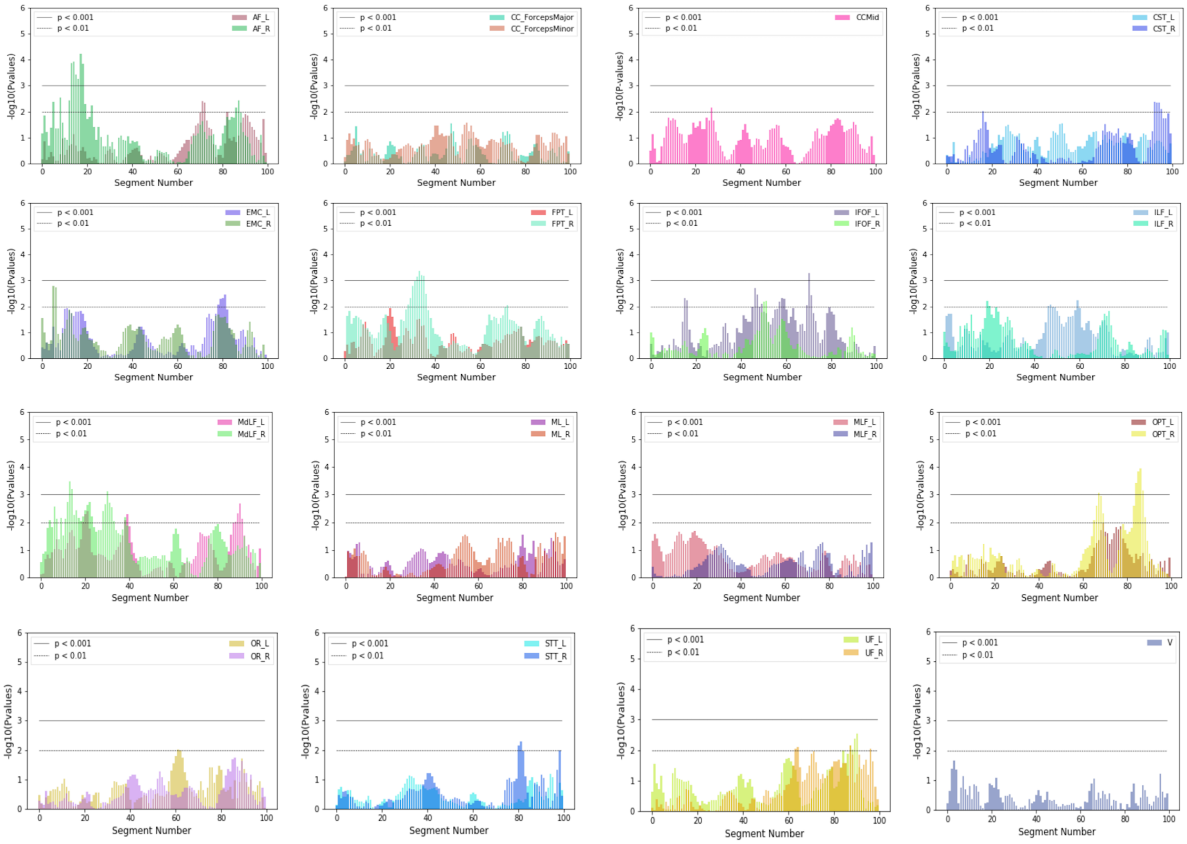

Figure A5. Plots summarizing population differences for CSA - generalized fractional anisotropy (CSA-GFA) using linear mixed models for 30 bundles along their length. On the x-axis, we have segment numbers and on the y-axis, we have negative logarithms of p-values. Here, the horizontal lower line indicates a p-value < 0.01 and the horizontal upper line indicates a p-value < 0.001. The p-value at a specific segment implies how much significant CSA-GFA group differences are there between patients and healthy controls for that particular bundle.

Fig. A5, shows LMM result plots of CSA-GFA measure. We found significant group differences AF_L, AF_R, CST_L, CST_R, EMC_L, FPT_L, FPT_R, IFOF_L, IFOF_R, ILF_R, MdLF_L, MdLF_R, OPT_R, OR_R, UF_L AND UF_R bundles.

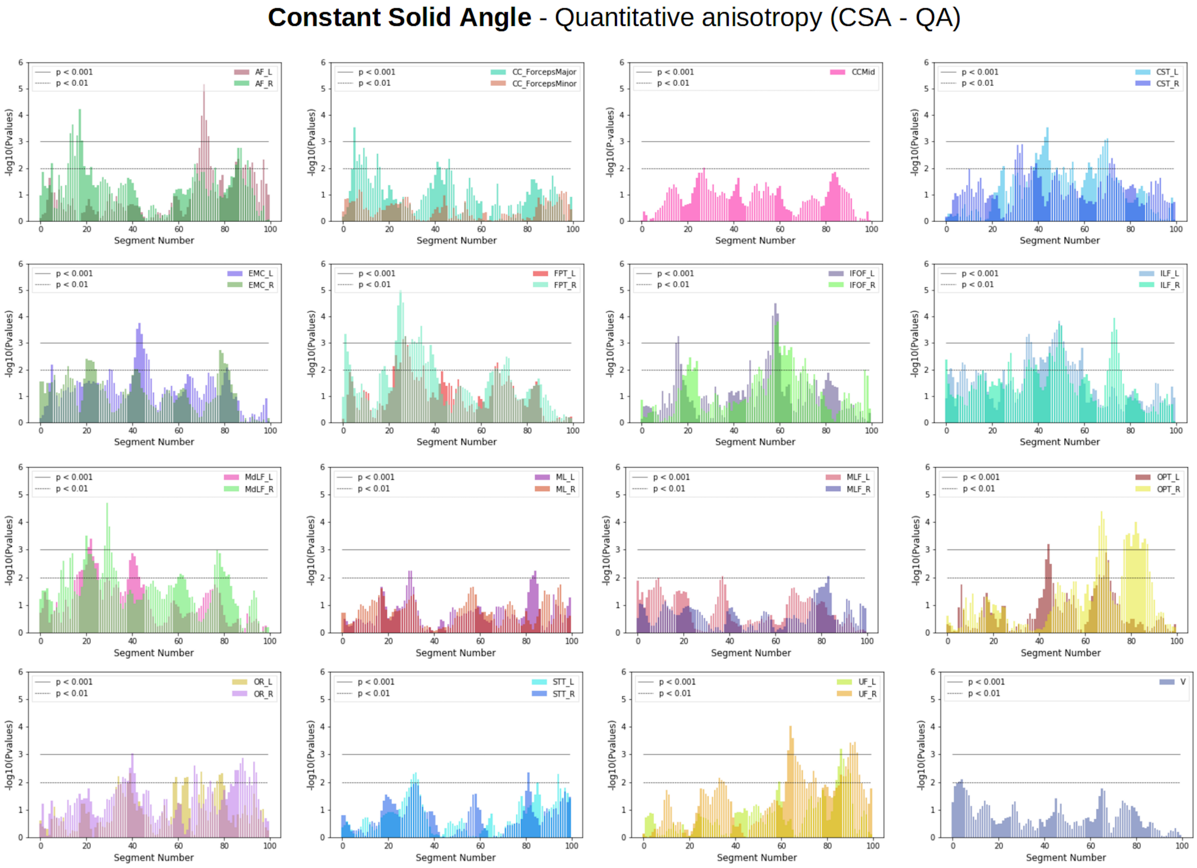

Figure A6. Plots summarizing population differences for CSA - quantitative anisotropy (CSA-QA) using linear mixed models for 30 bundles along their length. On the x-axis, we have segment numbers and on the y-axis, we have negative logarithms of p-values. Here, the horizontal lower line indicates a p-value $<$ 0.01 and the horizontal upper line indicates a p-value $<$ 0.001. The p-value at a specific segment implies how much significant CSA-QA group differences are there between patients and healthy controls for that particular bundle.

Fig. A6, shows LMM result plots of CSA-QA measure. We found significant group differences AF_L, AF_R, CST_L, CST_R, EMC_L, FPT_L, FPT_R, IFOF_L, IFOF_R, ILF_L, ILF_R, MdLF_L, MdLF_R, OPT_L, OPT_R, OR_R, UF_L AND UF_R bundles.

Appendix A.3 Hierarchical Clustering of BA Scores

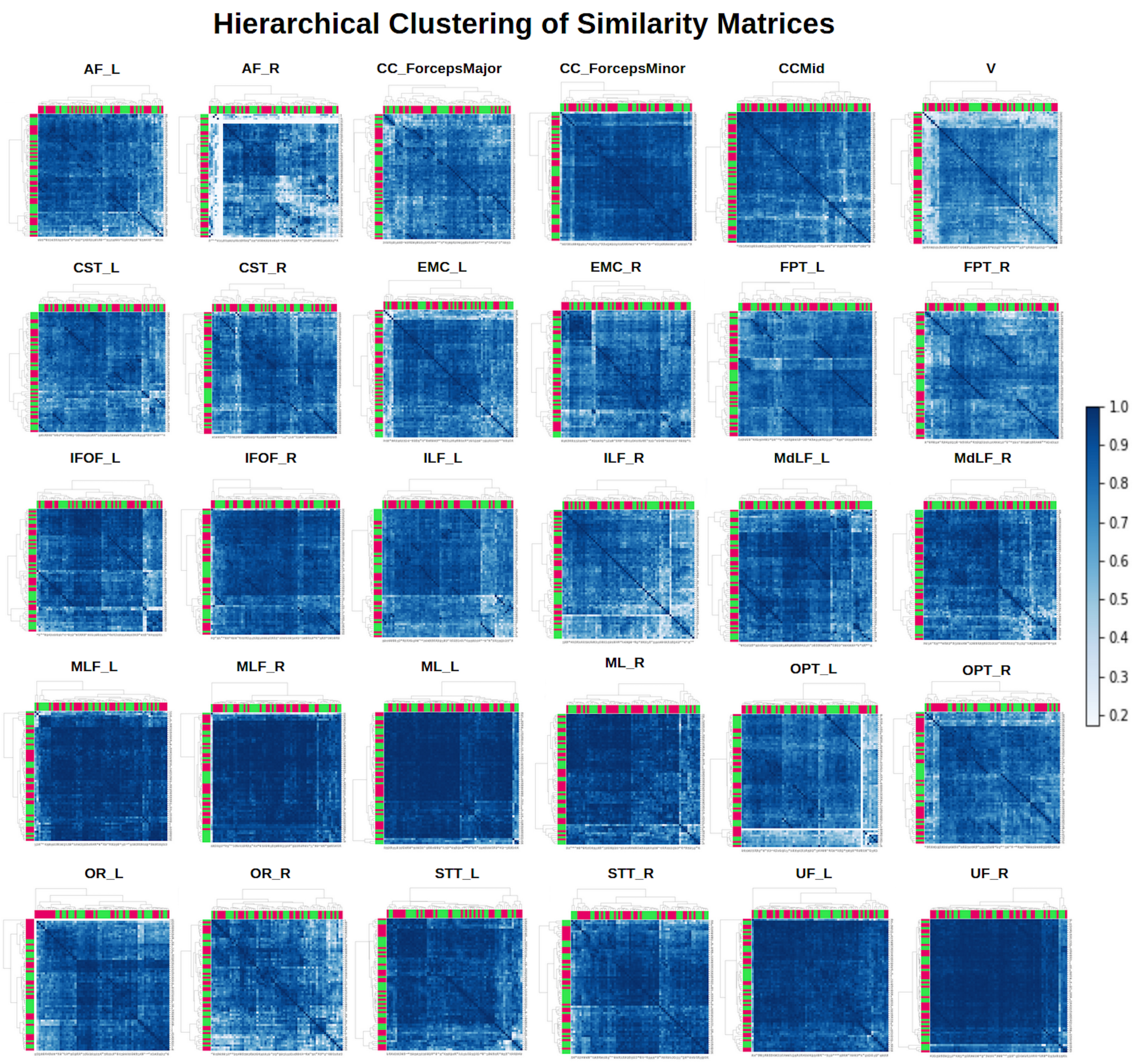

Figure A7. Hierarchical clustering of bundle adjacency similarity scores matrices. Subjects 0-31 belong to control group and subjects 32-63 belong to patients. The red color label represents patient subject and the green color label represents control subject.

We applied Ward's hierarchical clustering to the 30 similarity matrices generated from bundle adjacency (BA) method. Each matrix is a 64×64 matrix. Fig. A7, shows the results of clustering on the 30 similarity matrices of 30 bundles.

Appendix A.4 Linear Mixed Models

Linear mixed-effects models[9,10,11] are extensions of linear regression models for data that are clustered. These models describe the relationship between a response variable and independent variables, with coefficients that can vary with respect to one or more covariates. A mixed effects model has both random and fixed effects while a standard linear regression model has only fixed effects. The incorporation of random effects account for the correlation among repeated measures on the same individual or cluster. A fixed effect is an effect that is constant for a given population. A random effect is an effect that varies for a given population (it may be constant within sub populations but varies within the overall population). Linear mixed models (LMM) is defined as:

$$y = X\beta + Zu + \varepsilon$$

Where,

- y is the outcome variable, a $N \times 1$ column vector
- X is a $N \times p$ matrix of the p predictor variables
- β is a $p \times 1$ column vector of the fixed-effects regression coefficients (the βs)
- Z is the $N \times q$ design matrix for the q random effects, columns of Z are usually subset of the columns of X, q<p.
- u is a $q \times 1$ vector of the random effects, $u \sim$ N(0,D)
- ε is a $N \times 1$ column vector of the error, $\varepsilon \sim$ N(0,R)

In our case, the fixed effects term is accounted for population mean and random effects coefficients account for subject specific deviation from population mean. Random effects models are used to properly account for the correlations between points on the same streamline belonging to one segment for a specific subject. The response variable will be the anatomical measure such as FA, MD, RD, AD, CSA-GFA, and CSA-QA.

Appendix A.5 BUAN Processing Time

We report time taken by each step in the BUAN framework on a system with 32 GB RAM, and one Intel Core i7-7700K CPU with 8 cores.

BUAN Processing Time Report			
**Step**	**Method**	**Data**	**Time in Hours**
1	Streamline-based Registration (SLR)	64 subjects	2.211
2	RecoBundles (RB)	30 Bundles for 64 Subjects	23.179
3	Assignment Map	30 Bundles for 64 Subjects	0.174
4	Saving assignment maps information and anatomical measures in HDF5 files	30 Bundles of 64 subjects, 6 measures	5.07
5	Linear mixed Models (LMM)	30 Bundles of 64 subjects, 6 measures	14.629
6	Bundle Adjacency (BA)	30 bundles, 64 subjects with each other	16.213

Figure A8. Time taken by each step in the BUAN framework for analysis of 64 subjects. Steps from 1 to 4 depend on the output of the previous steps but step 5 and 6 are independent of each other. Hence, total time taken by the BUAN framework was 46.847 hours.

Since step 5 and 6 are independent of each other and can be computed in parallel, the total time taken by BUAN framework for 64 subjects is 46.847 hours. Here, we are reporting timings on a single machine. Clearly, the BUAN framework will be much faster when computed on supercomputers or using the cloud.

Appendix A.6 Cost Functions

Appendix A.6.1. ***Minimum Direct Flip***

The minimum average direct-flip (MDF)[12] distance is used for calculating distance between two streamlines that have same number of points. It is defined as follows: Let s and t be two streamlines with same number of points.

$$d_{direct}(s,t) = d(s,t)$$

$$d(s,t) = \frac{1}{k}\sum_{i=1}^{K} |s_i - t_i|$$

$$d_{flipped}(s,t) = d(s,t^F) = d(s^F,t)$$

$$MDF(s,t) = min(d_{direct}(s,t), d_{flipped}(s,t))$$

Appendix A.6.2. ***Bundle Minimum Distance***

The bundle-based minimum distance (BMD) [13] is used for calculating distance between two bundles (two sets of streamlines). The BMD is defined as follows: Let B_1 and B_2 be bundles where n_1 and n_2 represent total number of streamlines in each bundle respectively. And b_{1i} represents i^{th} streamine in bundle B_1 and b_{2j} represents j^{th} streamine in bundle B_2. A rectangular matrix D populated with all pairwise MDF streamline distances. Every element of D matrix is calculated by taking MDF distance of point p of b_{1i} streamline of B_1 bundle with point p of b_{2j} streamline of B_2 bundle, $D_{i,j} = MDF(b^p_{1i}, b^p_{2j})$. The most similar streamlines from one bundle to the other are given more weightage by MDF values of the rows and columns of the D matrix.

$$BMD(B_1,B_2) = \frac{1}{2}\left(\frac{1}{n_1}\sum_{i=1}^{n_1} \min_j D(i,j) + \frac{1}{n_2}\sum_{j=1}^{n_2} \min_i D(i,j)\right)^2$$

References

[1] Yeh, F.-C. *et al.* Population-averaged atlas of the macroscale human structural connectome and its network topology. *NeuroImage* **178**, 57–68 (2018).

[2] Garyfallidis, E. *et al.* Recognition of white matter bundles using local and global streamline-based registration and clustering. *NeuroImage* (2017).

[3] Garyfallidis, E. *et al.* Dipy, a library for the analysis of diffusion mri data. *Frontiers in Neuroinformatics* **8** (2014).

[4] Basser, P. J. & Pierpaoli, C. Microstructural and physiological features of tissues elucidated by quantitative-diffusion-tensor mri. *Journal of magnetic resonance, Series B* **111**, 209–219 (1996).

[5] Descoteaux, M. High angular resolution diffusion imaging (HARDI). *Wiley Encyclopedia of Electrical and Electronics Engineering* 1–25 (1999).

[6] Aganj, I. *et al.* Reconstruction of the orientation distribution function in single-and multiple-shell q-ball imaging within constant solid angle. *Magnetic resonance in medicine* **64**, 554–566 (2010).

[7] Tuch, D. S. Q-ball imaging. *Magnetic Resonance in Medicine: An Official Journal of the International Society for Magnetic Resonance in Medicine* **52**, 1358–1372 (2004).

[8] Yeh, F.-C., Wedeen, V. J. & Tseng, W.-Y. I. Generalized q-sampling imaging. *IEEE transactions on medical imaging* **29**, 1626–1635 (2010).

[9] Laird, N. M., Ware, J. H. *et al.* Random-effects models for longitudinal data. *Biometrics* **38**, 963–974 (1982).

[10] Hedges, L. V. A random effects model for effect sizes. *Psychological Bulletin* **93**, 388 (1983).

[11] Verbeke, G. & Molenberghs, G. *Linear mixed models for longitudinal data* (Springer Science & Business Media, 2009).

[12] Garyfallidis, E., Brett, M., Correia, M. M., Williams, G. B. & Nimmo-Smith, I. Quickbundles, a method for tractography simplification. *Frontiers in neuroscience* **6**, 175 (2012).

[13] Garyfallidis, E., Ocegueda, O., Wassermann, D. & Descoteaux, M. Robust and efficient linear registration of white-matter fascicles in the space of streamlines. *NeuroImage* **117**, 124–140 (2015).
